# Morphological re-description and molecular identification of Tabanidae (Diptera) in East Africa

**DOI:** 10.3897/zookeys.769.21144

**Published:** 2018-06-26

**Authors:** Claire M. Mugasa, Jandouwe Villinger, Joseph Gitau, Nelly Ndungu, Marc Ciosi, Daniel Masiga

**Affiliations:** 1 International Centre of Insect Physiology and Ecology (icipe), Nairobi, Kenya; 2 School of Biosecurity Biotechnical Laboratory Sciences, College of Veterinary Medicine, Animal Resources and Biosecurity (COVAB), Makerere University Kampala, Uganda; 3 Social Insects Research Group, Department of Zoology and Entomology University of Pretoria, Hatfield, 0028 Pretoria, South Africa; 4 Institute of Molecular Cell and Systems Biology, University of Glasgow, Glasgow, UK

**Keywords:** Tabanids, biting flies, morphology, cytochrome c oxidase 1, *COI*, Kenya, Uganda, Tanzania

## Abstract

Biting flies of the family Tabanidae are important vectors of human and animal diseases across continents. However, records of Africa tabanids are fragmentary and mostly cursory. To improve identification, documentation and description of Tabanidae in East Africa, a baseline survey for the identification and description of Tabanidae in three eastern African countries was conducted. Tabanids from various locations in Uganda (Wakiso District), Tanzania (Tarangire National Park) and Kenya (Shimba Hills National Reserve, Muhaka, Nguruman) were collected. In Uganda, octenol baited F-traps were used to target tabanids, while NG2G traps baited with cow urine and acetone were employed in Kenya and Tanzania. The tabanids were identified using morphological and molecular methods. Morphologically, five genera (*Ancala, Tabanus, Atylotus, Chrysops* and *Haematopota*) and fourteen species of the Tabanidae were identified. Among the 14 species identified, six belonged to the genus *Tabanus* of which two (*T. donaldsoni* and *T. guineensis*) had not been described before in East Africa. The greatest diversity of tabanid species were collected from the Shimba Hills National Reserve, while collections from Uganda (around the shores of Lake Victoria) had the fewest number of species. However, the *Ancala* genus was found in Uganda, but not in Kenya or Tanzania. Maximum likelihood phylogenies of mitochondrial cytochrome c oxidase 1 (*COI*) genes sequenced in this study show definite concordance with morphological species identifications, except for *Atylotus*. This survey will be critical to building a complete checklist of Tabanidae prevalent in the region, expanding knowledge of these important vectors of human and animal diseases.

## Introduction

Biting flies of the family Tabanidae (Order Diptera) are of both medical and veterinary importance because the females of most species are blood feeders that can transmit various pathogens to hosts as they feed on animals and humans (Foil 1989, Waage 1949). Pathogens transmitted by Tabanidae include bacteria, protozoa, helminths and viruses (Foil 1989). Moreover, because of their stout mouthparts, tabanids inflict painful bites while feeding, which affects livestock production as the animals are distracted from feeding, resulting in reduced growth rates, weight gain, reduced milk production, and reduced drought resistance, among others. The bite site may also predispose the animal to secondary infections, resulting in loss of hide quality (Yagi 1968).

Because different tabanid genera have been implicated as vectors of various pathogens, their accurate identification is important for disease ecology and management. The role of tabanids in the transmission of arboviruses such as Bovine Leukaemia Virus (BLV) has been documented (Monti et al. 2007). The potential for mechanical transmission of pathogens was explored by Buxton et al. (1985) and Foil et al. (1988). Buxton and colleagues investigated the ability of tabanids to transmit BLV in an experimental set-up. Using capillary action, infected blood was applied to the mouthparts of *Chrysops* spp. and *Tabanus atratus* Fabricius, 1775, which were removed to create inoculum that infected two sheep (Buxton et al. 1985). Similarly, experiments by Foil et al. (1988) demonstrated BVL transmissibility of groups of 50 and 100 horseflies (*Tabanus fuscicostatus*) from a cow with an infectious viral titre between 10^3^ and 10^4^ doses per ml. Likewise, the role of genus *Tabanus* in the horizontal transmission (transfer from one host to another) of BLV has been reported (Manet et al. 1989). Further, there are reports on the mechanical transmission of *Trypanosoma congolense* (Desquesnes and Dia 2003) and *Trypanosoma vivax* (Desquesnes and Dia 2004) by tabanids of different genera, including *Atylotus*. Indeed, tabanids are the principal vectors of *T. vivax* outside the sub-Saharan Africa tsetse fly belt (Gardiner and Wilson 1987). The genus *Chrysops* is reported to vector *Francisella tularensis* bacteria that cause tularemia in temperate regions, while in the tropics this genus is known to transmit the filarial nematode, *Loa loa*, in many sub-Saharan countries, including Uganda (Duke 1955). Therefore, effective control of diverse vectored diseases can be aided by accurate identification of tabanids in East Africa, which is crucial for monitoring their potential for invasion into naïve ecosystems and for vector control strategies.

To date, identification of tabanids is based on morphological keys and literature that was published over half a century ago (Oldroyd 1954). At present, the description of the tabanid fauna of eastern Africa is fragmented and sparsely documented without final valid checklists. The lack of concrete surveys and documentation of East African tabanids since 1954 constrains investigations of their ecology, zoogeography, vector-host interactions and importance as disease vectors.

Given the limited information on the taxonomy of these flies, their precise identification and classification is currently virtually impossible. This hurdle can be better overcome with the use of molecular DNA barcoding approaches (Hebert et al. 2003; Ratnasingham and Hebert 2007). Indeed, DNA barcoding based on the mitochondrial cytochrome c oxidase subunit 1 (*COI*) gene sequences has been used to discover new or previously unknown biodiversities due to its ability to differentiate diverse arthropod species including insect pests such as tussock moths, Lepidoptera: Lymantriidae (Ball and Armstrong 2005), mayflies (Ephemeroptera) (Ball et al. 2005) and spiders (Barret and Hebert 2005). More recently, Morita et al. (2016) successfully revised the phylogenetic framework of Tabanidae by employing four sets of genes including the mitochondrial *COI* on a data set of 110 horsefly species.

This study was undertaken to describe, identify and document a baseline *COI* barcode record of tabanid species occurring at diverse locations in Kenya, Uganda and Tanzania using morphological identifications to support a baseline *COI* barcode record.

## Methods

### Fly collection sites

**Figure 1.**
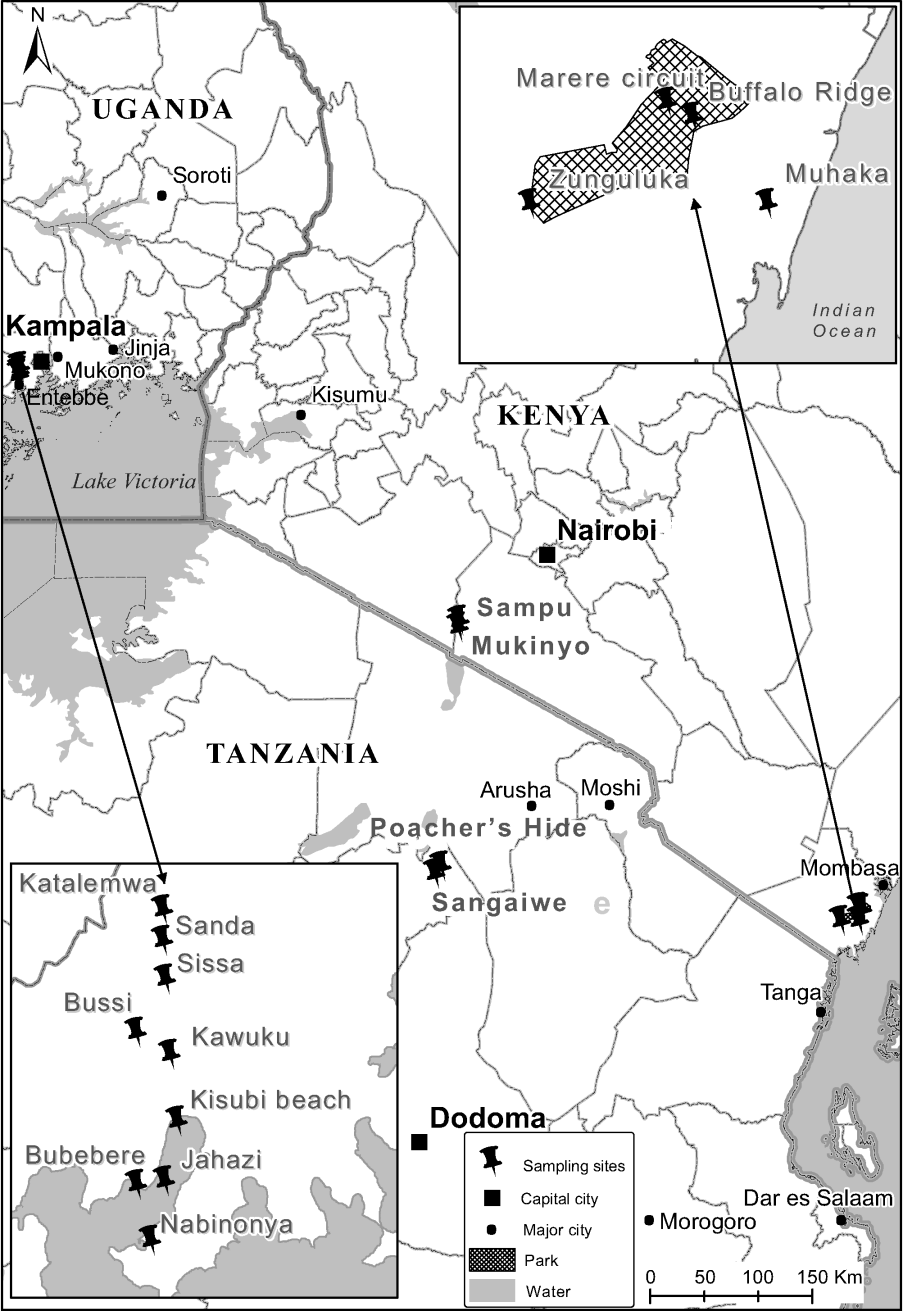
Fly collection sites in East Africa. In Uganda, collection sites were concentrated around the Lake Victoria basin close to swamps; in Kenya, there were two study regions one being more inland while the other was closer to the coast; in Tanzania flies were collected more inland.

In Uganda, flies were trapped in the Lake Victoria basin, specifically in Wakiso District from various sites (Table 1 and Fig. 1), between March and May 2013. Collection efforts were concentrated primarily around the swampy areas of the shores of Lake Victoria scattered with short shrubs, except for a few sites as indicated in Table 1.

**Table 1.** Fly collection sites and their respective GPS coordinates.

Country	Site name	Latitude	Longitude	Elevation (m)	Exact Collection sites
Uganda	Bubebere	0°5'1.968"N, 32°25'39.4392"E		1136	Swamp at Lake shore
	Jahazi	0°5'37.212"N, 32°26'20.9256"E		1140	Swamp at Lake shore
	Kisubi Beach	0°7'9.984"N, 32°26'58.5672"E		1137	Swamp at Lake shore
	Nabinonya	0°4'13.084"N, 32°28'46.9416"E		1135	Swamp at Lake shore
	Katalemwa	0°13'59.52"N, 32°26'30.2928"E		1154	Away from open water
	Sanda	0°13'0.516"N, 32°26'30.0552"E		1157	Away from open water
	Sissa	0°11'43.692"N, 32°26'35.7756"E		1144	Away from open water
	Kawuku	0°9'17.064"N, 32°26'44.8008"E		1175	Away from open water
	Bussi	0°9'59.904"N, 32°25'38.2044"E		1172	Away from open water
Kenya	Sampu	1°53'23.1108"S, 36°4'26.2452"E		663	Conservation/tourist area
	Mukinyo	1°50'2.778"S, 36°4'59.3508"E		672	Conservation/tourist area
	Marere Circuit	4°13'36.1164"S, 39°24'46.116"E		390	Conservation/tourist area
	Zunguluka	4°20'11.9472"S, 39°15'52.1892"E		137	Conservation/tourist area
	Buffalo Ridge	4°14'29.9724"S, 39°26'18.0348"E		367	Conservation/tourist area
	Muhaka	4°19'51.1284"S, 39°31'16.8492"E		663	Conservation/tourist area
Tanzania	Sangaiwe	3°56'29.76"S, 35°52'45.768"E		1000	National Park
	Poachers’ Hide	3°52'57.792"S, 35°56'1.428"E		999	National Park

In Kenya, flies were collected in four sites in the Shimba Hills National Reserve (Buffalo Ridge, Marere Circuit) and the environs in Zunguluka and Muhaka in August 2012. The Buffalo Ridge and Marere Circuit are in designated conservation areas where human activity is limited. Flies were also collected in two sites (Sampu and Mukinyo) in the Nguruman conservation area of southern Kenya in August 2012. The area is characterised by short grasslands interspersed with trees. In Tanzania, flies were collected from two sites (Sangaiwe and Poachers Hide) in the Tarangire National Park in August 2013.

### Trapping and preservation of flies

In Uganda, the F-traps (Kuzoe and Schofield 2005) baited with 1,8-octenol were deployed in the various sites selected depending on proximity to grazing land and swampy area or close to running water for 3 to 5 days depending on the fly density. In Kenya and Tanzania, tabanids trapped corresponded to incidental trapping as part of a tsetse fly trapping campaign using NG2G traps baited with less than 3 weeks old cow urine and acetone (Brightwell et al. 1987). All flies from Kenya and Tanzania were stored in 95% ethanol and transported to *icipe* at room temperature, while flies collected from Uganda were stored in 70% ethanol in a cool box.

### Morphological re-description of flies

**Figure 2.**
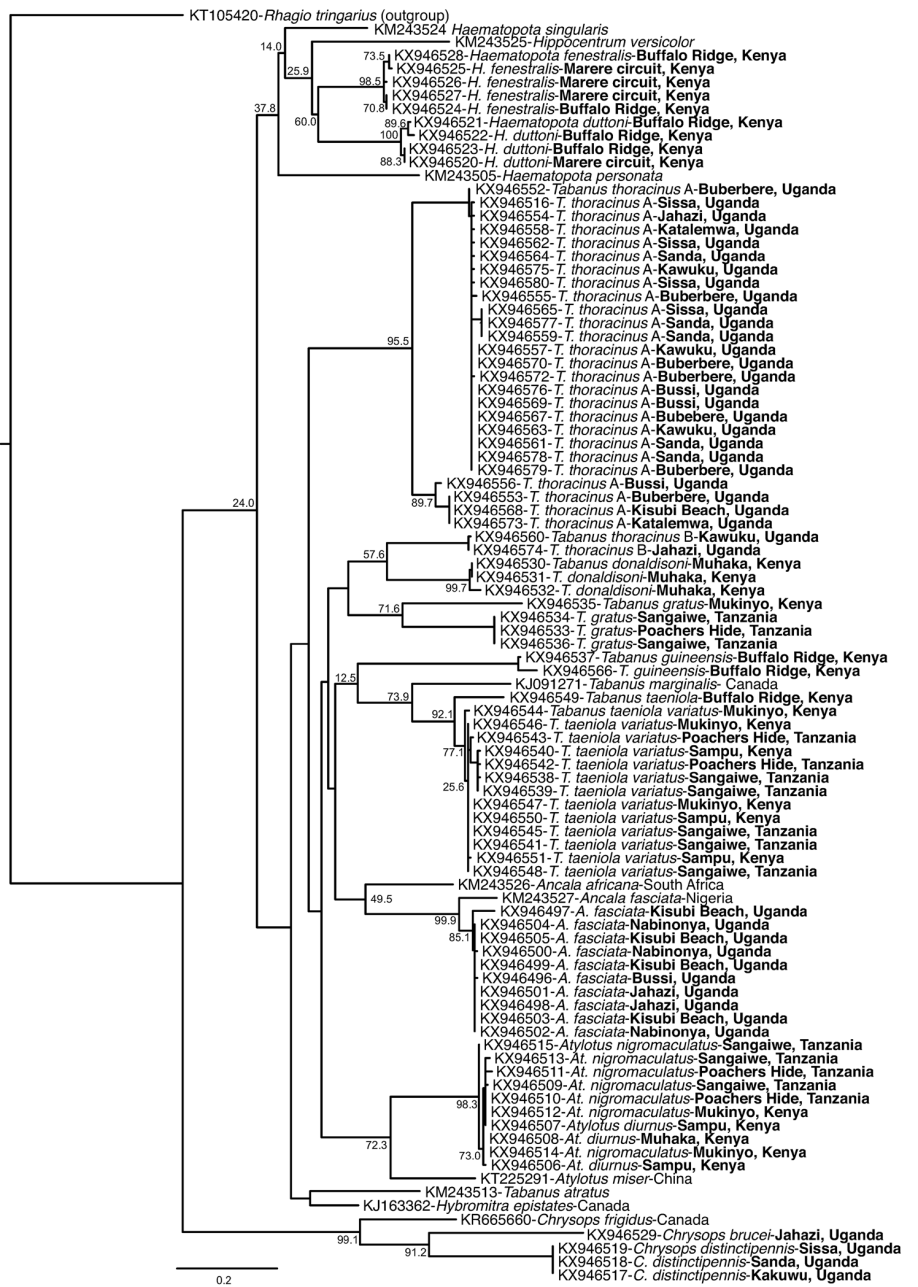
Maximum likelihood phylogenetic tree of *TabanidaeCOI* sequences. Sequences from samples collected in Kenya, Tanzania and Uganda alongside reference sequences obtained from GenBank. GenBank accessions, tabanid species and sampling locations (where available) are shown. Study sequences are indicated with sampling sites in bold. The branch length scale bar indicates nucleotide substitutions per site. Bootstrap values at the major nodes are of percent agreement among 1000 replicates.

**Figure 3.**
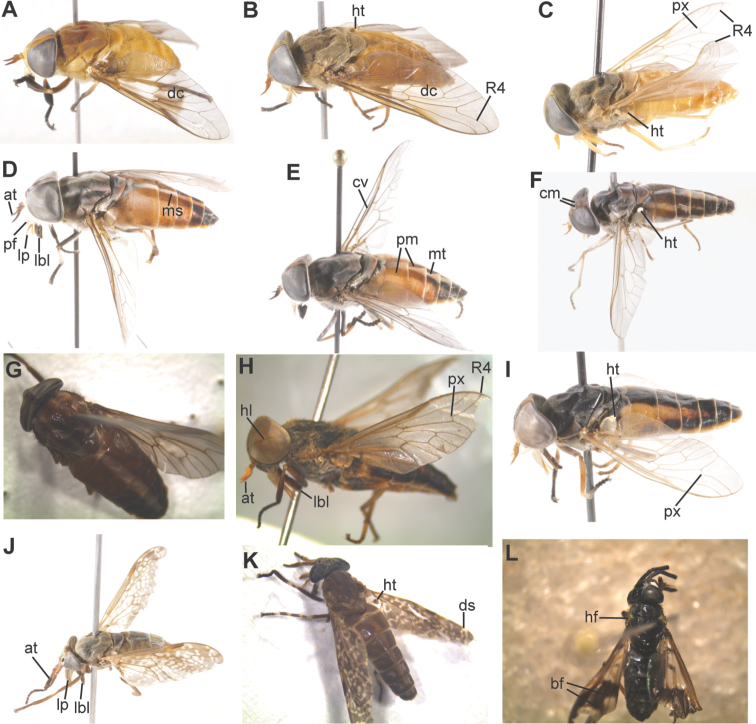
Whole tabanid flies. **A** *Ancala fasciata*, discal cell (dc) **B** *Tabanus thoracinus*, discal cell (dc), haltare (ht) **C** *Tabanus donaldsoni*, distinct R4 appendix (px) **D** *Tabanus taeniola*, median stripe (ms), parafacial hair (pf), antennae (at), labellum (lbl), palpus (lp) **E** *Tabanus taeniola variatus*, medial triangles (mt), peri-median bands (pm) **F** *Tabanus gratus*, haltares (ht), comma shaped shades (cm) **G** *Tabanus guineensis* **H** *Atylotus nigromaculatus*, eyes with thin black horizontal line (hl) **I** *Atylotus diurnus*, haltares (ht), wing with indistinct appendix (px) **J** *Haematopota duttoni* **K** *Haematopota fenestralis*, double white streak (ds) **L** *Chrysops distinctipennis*, hair tufts (hf), bifurcating band (bf).

**Figure 4.**
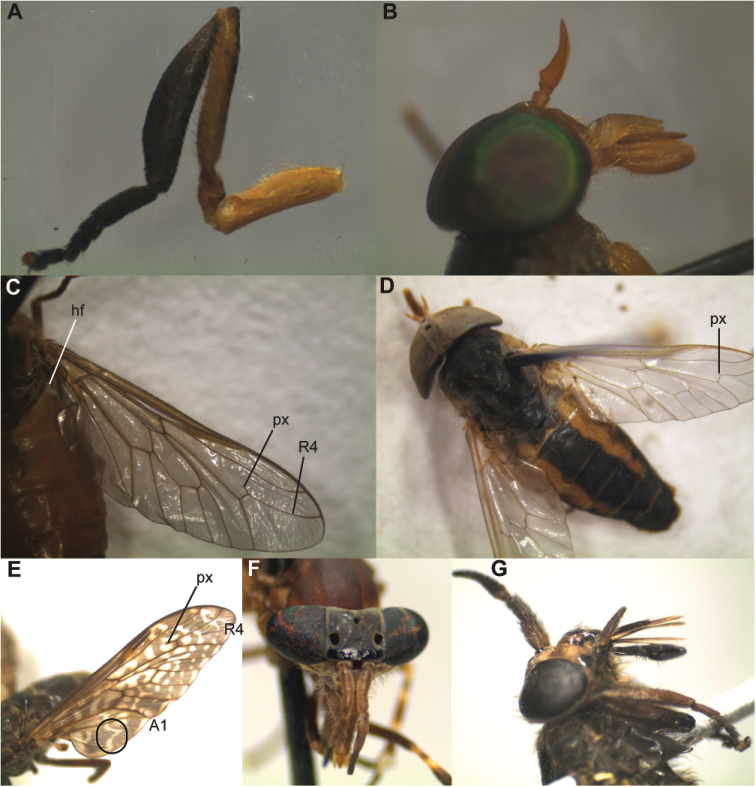
Key morphological features of sampled tabanid species**. A** *Ancala fasciata* **B** *Tabanus donaldsoni* green eyes (in fresh sample) **C** *Tabanus donaldsoni*, wing with distinct R4 appendix (px), indistict hair tuft (hf) **D** *Atylotus nigromaculatus*, wing with short, clear appendix on R4 **E** *Haematopota duttoni*, mottled wing with a right angled white thick line (in black circle) between vein A1 and the wing margin, the R4 has a long appendix **F** *Haematopota fenestralis*, shiny black callus on frons, eyes have bright coloured bands **G** *Chrysops brucei*, lateral view of head showing the antenna, black mouth parts and brown legs (partial).

Flies were pinned and placed in entomological boxes for morphological identification and labelled with area and date of collection. Entomological boxes were kept in the Biosystematic Support Unit (BSU) in *icipe*. Subsequently, the flies were examined under a light microscope at a magnification of X10; for finer details, a higher magnification of X40 was used. The morphological keys used were those documented by Oldroyd (1952, 1954, 1957). After morphological identification, images of selected specimens were taken using a Nikon D90 camera for the gross specimen and Leica EZ4D to capture finer details. Images of the gross specimen are presented as Figs 2–4 as well as Suppl. material 2: Figures S1–S13.

### Molecular characterisation of flies

From each group of morphologically identical specimens, a tibia was isolated from at least one fly in each group (with similar morphological features) and placed in separate plate-wells with 30 µl of 70% ethanol each. Two hundred and nine tissue samples were submitted for DNA extraction and *COI* barcode sequencing to the Canadian Centre for DNA Barcoding, University of Guelph, out of which 84 sequences could be generated (Suppl. material 1). An image of the dorsal and ventral side of the whole fly was also submitted for each tissue sample. For assessment of taxonomic relationships, the *COI* mitochondrial gene was targeted to generate a DNA fragment of about 648-bp (Ratnasingham and Hebert 2007). The verified barcode sequences associated with specimen metadata and pictures in the Barcode of Life Database (BOLD) were submitted to GenBank (accessions KX946496–KX946580).

### Phylogenetic analysis

We used MAFFT (Katoh and Standley 2013) within Geneiousv8.1.4 (available from http://www.geneious.com), software created by Biomatters (Kearse et al. 2012), to align all sequences alongside related tabanid sequences identified by querying against the GenBank nr database (http://www.ncbi.nlm.nih.gov/) using the Nucleotide Basic Local Alignment Search Tool (BLASTn) (Altschul et al. 1990), with *Rhagio tringarius* (GenBank accession KT105420) as an outgroup. Using PhyML v. 3.0 (Guindon et al. 2010), we inferred a maximum likelihood phylogeny from this alignment. The phylogeny employed the Akaike Information Criterion for automatic model selection and tree topologies were estimated using nearest neighbor interchange (NNI) improvements over 1000 bootstrap replicates. A midpoint rooted phylogenetic tree was then generated from the phylogeny using FigTree v1.4.2 (Drummond and Rambaut 2007).

## Results

From Uganda, 995 female tabanids were collected, while approximately 2300 female tabanids were collected in both Kenya and Tanzania. Male flies were collected in negligible numbers and where thus not included in the study. The tabanids collected were grouped based on both morphology and *COI* barcode sequence phylogenies (Fig. 2) into five genera (*Tabanus*, *Ancala*, *Atylotus, Haematopota* and *Chrysops*) comprising 12 species as shown in Table 2.

**Table 2.** Samples of each morphological identification that were submitted for sequencing from each locality per country.

Country	Site of collection	Genus	Species	No. Submitted	No. Sequenced
Kenya	Buffalo Ridge	*Haematopota*	*H. duttoni*	5	3
			*H. fenestralis*	4	2
		*Tabanus*	*T. taeniola*	1	1
			*T. guineensis*	2	2
	Mukinyo	*Tabanus*	*T. taeniola variatus*	3	3
			*T. gratus*	1	1
		*Atylotus*	*At. nigromaculatus*	4	2
	Marere Circuit	*Haematopota*	*H. fenestralis*	3	3
			*H. duttoni*	1	1
	Zunguluka	*Haematopota*	*H. fenestralis*	4	0
	Sampu	*Tabanus*	*T. taeniola variatus*	3	3
		*Atylotus*	*At. diurnus*	2	2
	Muhaka	*Tabanus*	*T. donaldsoni*	3	3
		*Atylotus*	*At. diurnus*	3	1
Tanzania	Sangaiwe	*Tabanus*	*T. taeniola variatus*	5	5
			*T. gratus*	5	2
		*Atylotus*	*At. nigromaculatus*	8	3
	Poacher’s Hide	*Tabanus*	*T. gratus*	1	1
			*T. taeniola variatus*	2	2
		*Atylotus*	*At. nigromaculatus*	2	2
Uganda	Mabamba	*Tabanus*	*T. thoracinus*	23	1
		*Chrysops*	*C. brucei*	1	0
		*Ancala*	*A. fasciata*	2	0
	Bussi	*Tabanus*	*T. thoracinus*	12	4
	Bubebere	*Tabanus*	*T. thoracinus*	37	6
	Bugogo	*Tabanus*	*T. thoracinus*	1	0
	Mikka	*Tabanus*	*T. thoracinus*	2	0
	Elubbe	*Tabanus*	*T. thoracinus*	3	0
	Nabinonya	*Ancala*	*A. fasciata*	2	2
	Kawuku	*Chrysops*	*C. distinctipennis*	1	1
		*Tabanus*	*T. thoracinus*	11	5
	Katalemwa	*Tabanus*	*T. thoracinus*	7	3
	Sissa	*Tabanus*	*T. thoracinus*	16	4
	Sanda	*Chrysops*	*C. distinctipennis*	1	1
		*Tabanus*	*T. thoracinus*	6	5
	Kisubi	*Ancala*	*A. fasciata*	4	4
		*Tabanus*	*T. thoracinus*	9	1
	Jahazi	*Ancala*	*A. fasciata*	4	3
		*Chrysops*	*C. brucei*	1	1
		*Tabanus*	*T. thoracinus*	4	1
**Total**	**209**	**85**			

### Molecular sequences of collected flies

Clusters within the maximum likelihood phylogeny of *COI* sequences (Fig. 2) corresponded closely with species’ identifications derived from morphological characters (below), except in the case of *T. thoracinus*, which formed two distinct clades designated as *T. thoracinus* A and *T. thoracinus* B (Fig. 2). DNA barcoding separated samples according to the species and the site of collection. The tree separates into two main branches representing the subfamilies Chrysopinae and Tabaninae; the latter further branches into the *Haematopota* species, *Tabanus*, *Ancala* and *Atylotus* species along with *Hippocentrum*, and *Hybomitra* reference sequences.

### Morphological description of tabanids collected

Images of the tabanids collected during this study and morphologically described at the genus and species level are presented in Figures 3 and 4 as well as in Suppl. material 2: Figure S1–S13.

We identified tabanid species based on their morphology and in reference to literature of Oldroyd (1952, 1954, 1957) as well as Morita (2008).

### Genus *Ancala*

#### Ancala fasciata

Taxon classificationAnimaliaDipteraTabanidae

Fabricus, 1775

Fig. 3A

##### Location.

Shores of Lake Victoria, Uganda

##### Descriprion.

Head. Head as wide as thorax (Suppl. material 2: Figure S1A). Eyes shiny green (in freshly collected specimen), turn black after preservation in ethanol. Eyes separated by narrow brown frons with brown and black short hair. Callus (cs) brown, wide basally and tapering posteriorly to form a spindle-shaped upper callus, reaching midway frons. Scar-like callus at posterior end of frons (Suppl. material 2: Figure S1B). Antennae brown, first antennal segment (scape) is yellowish golden-brown with predominantly white hair and few black hairs that appear as a thorn-like anterior projection as on second segment. Third segment (flagellum) brown, relatively wide, with small white hair and four dark brown annulations. Second segment of the palpus is yellowish white with white recumbent hair, interspersed with few black hairs. Palpus with black tip and black labellum (Suppl. material 2: Figure S1C).

Thorax. Thorax brown with brownish-black patterns and black and golden-brown hair, with light brown median stripe that runs down to posterior end of the mesonotum. Median sublateral stripes (st) light brown; may be obscured and only reach half way of mesonotum. Scutellum yellowish and brown shades (brown with yellow postero-lateral border) with white and black hair. Prominent white hair tuft (hf) below postalar callus at base of wing (Suppl. material 2: Figure S1D). Halteres whitish yellow with a yellow stalk. Wing with typical basicosta and costa covered with short black hair with a longitudinal hairless groove. Wing with brown longitudinal cross band that runs up to discal cell and does not reach hind margin. The discal cell (dc) brown (Suppl. material 2: Figure S1A). Fore tibia differs in the species, “swollen” and black with black hair (Fig. 4A; Suppl. material 2: Figure S1E). Fore femur is brown with black and white hair, tarsus black with black hair. Hind tibia brown with a darker shade distally and lighter basally. Distally hair predominantly black with few white hair, proximal more white hair. Hind femur brownish yellow or golden yellow with white hair, tarsus dark brown with black hair. Middle leg same as hind leg.

Abdomen. Abdomen yellow with yellow and black hair. Seventh segment pointed (Suppl. material 2: Figure S1A). Ventral surface yellow with yellow hair except on last segment with white and black hair.

### Genus *Tabanus*

#### Tabanus donaldsoni

Taxon classificationAnimaliaDipteraTabanidae

Carter, 1912

Fig. 3C

##### Location.

Muhaka, Kenya.

##### Descriprion.

Head. Head wider than thorax. Eyes dark green in freshly collected insects (Fig. 4C, Suppl. material 2: Figure S3A), but turn black after storage and separated by brown narrow frons with black and white hair. Callus brown; lower callus round-oval and projects upward in form of thick line (Suppl. material 2: Figure S3B). Antennae golden brown (typical of the genus *Tabanus*). Scape with white hair at base with black hair anteriorly. Small pedicel with short white hair all over and black hair only at anterior border. Palpus (lp) yellowish white with white and black hair; labellum golden brown (Suppl. material 2: Figure S3C).

Thorax. Thorax blackish brown with white and black hair. Median stripe not seen, sub-lateral stripes light brown and indistinct (only seen up to middle of thorax). Halteres yellowish brown. Wing clear and R4 on the wing has prominent appendix (px). Tufts of white hair (hf) near postalar callus indistinct (Fig. 4C, Suppl. material 2: Figure S3D). Fore tibia and fore femure yellowish brown, fore tibia covered with white and black hair, fore femur with only white hair. Fore tarsus light brown with black and white hair. Hind and middle legs similar to forelegs (Suppl. material 2: Figure S3E).

Abdomen. Golden-brown dorsal and ventral surfaces of abdomen without patterns but have black and white recumbent hair (Suppl. material 2: Figure S3A).

#### Tabanus gratus

Taxon classificationAnimaliaDipteraTabanidae

Loew, 1858

Fig. 3F

##### Location.

Tarangire National Park, Tanzania.

This fly is darker brown, smaller and slenderer than *T. taeniola* Palisot de Beauvois (1807). However, the colour of *T. gratus* Loew (1858) collected from Nguruman, Kenya is lighter brown with golden stripes on abdomen.

##### Descriprion.

Head. Head wider than thorax with slightly concave posterior vertex (Suppl. material 2: Figure S6A). Eyes black with green and black bands when fresh that disappear after the sample dries up. Eyes separated by dark brown frons with black hair. Basal callus dark brown, large and as wide as frons, extending upwards in slender projection connecting with smaller upper tear-drop shaped callus. On each side of upper callus a thick comma-shaped black shade. Long white parafacial hair (Suppl. material 2: Figure S6B). Scape large, light brown with white hair except at tip of the segment with black hair. Pedicel small and brown, more or less covered (obscured) between scape and flagellum, black hair at anterior end. Third segment orange brown with few black hairs. Second palpal segment whitish grey with black and white hair. Labellum brownish grey.

Thorax. Thorax black with black and brown hair; brown median stripe traverses thorax and lateral margins of the scutellum. Sub-lateral stripes greyish brown with golden brown hair. Lateral margins of thorax greyish white. Hair tufts only seen ventrally at the base of wing. White halteres (Suppl. material 2: Figure S6A). Brown fore tibia covered with short white and black hair except anteriorally dark brown covered with black hair. Fore femur brown with black hair and long white hair. Fore tarsus black with black hair. Hind tibia light brown with white and black hair. Hind femur light brown with long white hair. Tarsus yellowish brown with black and white hair. Middle legs same as hind legs. Wing clear and R4 has no appendix (Suppl. material 2: Figure S6C).

Abdomen. Abdomen brown, slender and tapers to the posterior end with black and brown hair, has white median and two lateral stripes pale brownish orange grey, with orange brown part at posterior end of each segment. Stripes have whitish hair. Median stripe narrow in first two segments but widens on the third, widest on the fourth segment before it tapering down to last segment. Lateral stripes wide on first and second segments but gradually narrow as they converge slightly at posterior end of abdomen. Lateral abdominal margins ashy grey (Suppl. material 2: Figure S6A). Ventral abdominal surface brown with white hair.

#### Tabanus guineensis

Taxon classificationAnimaliaDipteraTabanidae

Wiedemann, 1824

Fig. 3G

##### Location.

Shimba Hills, Kenya

Generally, the body is brown and slender (Suppl. material 2: Figure S7A).

##### Descriprion.

Head. Head black, as wide as thorax. Black eyes separated by black frons. Bi-partite callus dark brown joined by wide constricted neck and occupies more than half of frons (Suppl. material 2: Figure S7B). Antennae slender, golden-orange with black hair. Scape has prominent black anterior projection, as does pedicel. Projection on flagellum not prominent and annulations appear blackish. Second palpi white with black hair. Labellum greyish black.

Thorax. Brown thorax with two inconspicuous lateral greyish stripes. Thorax covered with recumbent black and white hair. Lateral thoracic margins grey with white hair. Scutellum brown with lighter shade posteriorly with brownish white hair especially at lateral margin. Yellowish brown hair tufts at postalar callus. Halteres light brown. Wing clear with brown pigment at the anterior margin; R4 has a short but visible appendix (Suppl. material 2: Figure S7C). Fore and hind tibia and femur brown with black hair.

Abdomen. Elongate with more or less parallel sides. Anterior abdominal segments light brown and become darker brown posteriorly with black and white hair. Abdomen bisected by a whitish median stripe with white hair. Last segment flat and rectangular (Suppl. material 2: Figure S7A).

#### Tabanus taeniola

Taxon classificationAnimaliaDipteraTabanidae

Palisot de Beauvois, 1807

Fig. 3D

##### Location.

Tarangire, Nguruman, Shimba Hills, Kenya

##### Descriprion.

Head. Head wider than thorax with long white parafacial hair (Suppl. material 2: Figure S4A). Eyes dark green in freshly collected specimen without any band; on preservation in ethanol, green colour is lost and eyes remain black. Palps with white hair; labellum black (Suppl. material 2: Figure S4B). Antennae brown and black (typical of genus *Tabanus*). Scape and pedicel orange-brown with black hair. Flagellum orange-brown at base and darkens towards dark brown to black annulations (Suppl. material 2: Figure S4B). Eyes separated by narrow pale brown frons with black and brown hair with brown bell-shaped callus that does not taper posteriorly (Suppl. material 2: Figure S4C).

##### Descriprion.

Thorax. Thorax greyish black with black and whitish hair and median whitish stripe almost reaching posterior end of thorax. Sub-lateral stripes (st) ashy grey, distinctly visible with white hair and continue to posterior mesonotum. Scutellum brown with indistinct hair. Hair tufts at postalar area whitish to golden brown. Halteres yellowish white (Suppl. material 2: Figure S4C). The distal 1/3 of fore tibia blackish brown with black hair, proximal 2/3 light brown/yellow. Fore femur greyish black with black and long silver-white hair and black tibia with black hair. Hind tibia (Suppl. material 2: Figure S4D) yellowish brown with black and white hair and hind femur greyish black with white hair; tarsus light brown with black hair. Middle leg similar to hind leg.

Abdomen. Abdomen orange brown and black, tapers posteriorly such that last segment appears pointed. Abdomen with two peri-median longitudinal orange rectangular bands that progressively darken to dark brown then to black on fifth and sixth segment. Peri-median longitudinal bands enclosing lighter coloured median stripe starts anteriorly as white, becomes orange then finally darkens to light brown on sixth segment. Seventh segment completely black (Suppl. material 2: Figure S4A). Ventro-medially, fine black recumbent hair that gives abdomen dark brown appearance.

#### Tabanus taeniola variatus

Taxon classificationAnimaliaDipteraTabanidae

Oldroyd, 1954

Fig. 3E

##### Location.

Tarangire National Park, Tanzania

##### Descriprion.

Head. Head wide, posterior vertex slightly concave (Suppl. material 2: Figure S5A). Eyes black without bands, separated by greyish frons with white hair and black hair posteriorly. Bell-shaped callus; dark brown basal callus joined to upper callus by narrow constriction (Suppl. material 2: Figure S5B). Distinct white parafacial hair as seen in *T. taeniola* described by Oldroyd (1954) (Suppl. material 2: Figure S4A), but with a different callus shape, where upper callus ends flat (not tapering), thus a different subspecies (*T. taeniola variatus*). Antennae with two shades of brown; scape is light brown with white hair at base and black hair at anterior. Anterior-most point of scapes has brownish orange tinge. Pedicel also light brown, small, appears engulfed between scape and flagellum segment with black hair anteriorly that appear as thorn-like projection. Flagellum light brown at base up to projection, but darkens anteriorly to dark brown; annulations black, similar to *T. taeniola* (Suppl. material 2: Figure S5A). Second segment of palpus dull white with white hair ventrally, dorsally few black hairs mixed among the white. Labellum black with black hair as described in *T. taeniola*.

Thorax. Thorax greyish brown with long whitish grey hair mixed with black hair. Median stripe distinctly whitish grey reaching posterior thorax. Sublateral stripes greyish white and less distinct, but nevertheless reach the posterior thorax. Dark brown scutellum with recumbent grey hair, especially at margins. Tufts of white hair at postalar callus wing base. Halteres whitish to golden yellow (Suppl. material 2: Figure S5B). Wing clear and R4 without appendix (Suppl. material 2: Figure S5C). Middle leg same as hind leg. Distal third (1/3) of fore tibia of foreleg is black with black hair; the rest of tibia light brown with white hair interspersed with few black hair (Suppl. material 2: Figure S5D). Brownish yellow hind tibia with white hair interspersed with few black hair, hind femur ashy grey with long white hair. Tarsus light brown with black hair (Suppl. material 2: Figure S5E) and each leg has two tarsal claws.

Abdomen. Abdomen golden brown with black and white hair, with dorso-medial black band on first two segments, broad on first segment and narrower on second. Band does not completely traverse second segment. Segments with distinct triangular pattern medially light brown anteriorly and fades into greyish brown posteriorly. Triangular patterns have black hair mixed with white hair. Abdomen with black hair laterally; medial abdomen greyish brown with white and black hair. Ventrally brown surface with whitish hair except for last two black segments (Suppl. material 2: Figure S5A).

#### Tabanus thoracinus

Taxon classificationAnimaliaDipteraTabanidae

Palisot de Beauvois, 1806

Fig. 3B

##### Location.

Shores of Lake Victoria, Uganda

Two genetic variants, that could not be distinguished on the basis of the morphological keys identified in the present study.

##### Descriprion.

Head. Head wider than the thorax (Suppl. material 2: Figure S2A) with yellowish white parafacial hair (pf). Eyes dark green (when freshly collected, before drying) without a band, separated by brown narrow frons with black and white hair. Antennae golden-brown (typical of *Tabanus*). Scape with white hair at base and black hair anteriorly, thus appearing to have a black tip (Suppl. material 2: Figure S2B). Small pedicel with short white hair all over and black hair only at anterior border. Callus (cs) brown; basal callus round-oval and projects upward tapering to form a thick black line (Suppl. material 2: Figure S2C). Palps yellowish white with white and black hair; labellum brown (Suppl. material 2: Figure S2B).

Thorax. Thorax blackish brown with white and black hair and invisible median stripe, sub-lateral stripes indistinct light brown only to middle of thorax. Wing clear and R4 does not have appendix (Suppl. material 2: Figure S2A). Fore tibia and fore femur yellowish brown; fore tibia covered with white and black hair, fore femur with white hair (Suppl. material 2: Figure S2D). Fore tarsus light brown with black and white hair. Hind and middle legs similar to forelegs. Halteres yellowish brown. Indistinct tufts of white hair near postalar callus.

Abdomen. Dorsal and ventral surfaces of abdomen orange-brown without patterns, have black and white recumbent hair. Ventral surface may have black hair concentrated on second last segment that appears as dark patch (Suppl. material 2: Figure S2A).

### Genus *Atylotus*

#### Atylotus nigromaculatus

Taxon classificationAnimaliaDipteraTabanidae

Ricardo, 1900

Fig. 3H

##### Location.

Tarangire National Park, Tanzania; Nguruman, Kenya.

Generally, slender body covered with dense golden-brown hair (Suppl. material 2: Figure S8A).

##### Descriprion.

Head. Head wide, posterior vertex slightly concave. Eyes pale golden brown with thin black horizontal line or band. Eyes separated by narrow golden-brown frons on posterior quarter and black anteriorly. Callus in two well-separated lower and upper parts shiny black and vaguely round (Suppl. material 2: Figure S8B). Antenna golden brown; Scapes with white hair and black hair anteriorly as does the pedicel. Pedicel less distinct as it closely adheres to much larger flagellum. Second palp segment white with white and black hair but only white hair seen ventrally; brown labellum (Suppl. material 2: Figure S8B).

Thorax. Black thorax with indistinct stripes and golden-brown hair (Suppl. material 2: Figure S8C). Indistinct hair tufts at postalar callus. Halteres yellowish white and clear wing; R4 with small appendix (Fig. 4D; Suppl. material 2: Figure S8A). Fore tibia black distally and light brown towards femur with black and white hair. Fore femur brown with grey patch at proximal end with white hair all over. Fore tarsus black with black hair (Suppl. material 2: Figure S8D). Hind tibia light brown with black and white hair, while hind femur light brown and proximally blackish grey with white hair all over the femur. Hind tarsus light brown with black and white hair (Suppl. material 2: Figure S8E); middle leg similar to hind leg.

Abdomen. Brown abdomen, slender and slightly tapers posteriorly. Broad black median band that longitudinally dissects abdomen; band with black recumbent hair (Fig. 4D). Brown patches with brown recumbent hair on either side of the band on first to fourth segment. Fifth to seventh segments black. Lateral abdominal margins black (Suppl. material 2: Figure S8C). Ventral surface of abdomen brown with white hair and dark grey margins.

##### Note.

*Atylotus nigromaculatus* is the only species in genus *Atylotus* described by Oldroyd (1954) whose eyes have no band. The morphological features described in this study are similar to features described by Taioe et al. (2017) of specimen collected from South Africa.

#### Atylotus diurnus

Taxon classificationAnimaliaDipteraTabanidae

Walker, 1850

Fig. 3I

##### Location.

Muhaka, Shimba Hills, Nguruman, all in Kenya.

Generally, the body is slender and not as hairy as in *At. nigromaculatus* described above.

##### Descriprion.

Head. Head wider than thorax and eyes pale black without band (Suppl. material 2: Figure S9A). Eyes separated by narrow frons brown on posterior quarter and black anteriorly with black and golden-brown hair. Basal callus brownish black and upper callus black. Two small calli vaguely rounded and well separated (Suppl. material 2: Figure S9B). Antenna yellowish orange; scape light yellow with white hair at base and black hair at anterior end as does the pedicel. Pedicel small with projection and is less distinct as it closely adheres to the much larger flagellum. Flagellum orange with blunt projection with small black hair. Second palp segment white with white and black hair; labellum dark brown (Suppl. material 2: Figure S9A).

Thorax. Thorax greyish black without visible median stripe, but lateral stripes black and indistinct, halteres white (Suppl. material 2: Figure S9A). Clear wing and R4 with short appendix (Suppl. material 2: Figure S9C). Legs as described in *At. nigromaculatus*.

Abdomen. Abdomen with thick black band running medially to last segment with black and few white hair; light brown part that is broader anteriorly but narrows posteriorly such that the sixth and seventh segments are completely black. Brown parts of abdomen with black and white hair, black part with mostly black hair and few white hairs. Lateral margins of abdomen black (Suppl. material 2: Figure S9A).

##### Note.

*Atylotus diurnus* is the only species described by Oldroyd (1952) whose wing has an appendix on the R4 of the wing. Though the other morphological features described here are different from those described by Oldroyds (1952), they are similar to those described by Taioe et al. (2017), thus confirming that the specimen are indeed *Atylotus diurnus*.

### Genus *Haematopota*

#### Haematopota duttoni

Taxon classificationAnimaliaDipteraTabanidae

Newstead, Dutton & Todd, 1907

Fig. 3J

##### Location.

Shimba Hills, Kenya.

##### Descriprion.

Head. Head wider than thorax with vertex at the posterior distinctly concave (Suppl. material 2: Figure S10A). Black eyes with brightly coloured zig-zag bands that fade on preservation. Eyes separated by wide frons; anterior third (1/3) of frons brown with brown and black hair, posteriorly blackish grey with whitish grey hair. Frontal callus shiny brown, wider than long, spanning entire width of frons. Pair of round dark brown purplish velvety spots above frontal callus at either side towards lateral margins of frons. Below frontal callus velvety dark brownish black sub callus. Below vertex of frons medially small black round velvety spot (Suppl. material 2: Figure S10B). Antennae typical of genus *Haematopota*; three segments dark brown with black and few short white hairs. Scape long and slightly wide, pedicel globular with anterior spike-like projection. Flagellum long and slender with three black annulations that have fine black hair. Second palpal segment elongate, light brown with white and black hair. Labellum dark brown (Suppl. material 2: Figure S10C).

Thorax. Thorax greyish brown with black and white hair. Median and sub lateral stripes whitish and run down to the posterior end of thorax. Fore tibia laterally flattened and dark brown with black and (few) white hair. Fore femur dark brown with black hair. Hind tibia dark brown with black and white hair; hind femur dark brown with black hair. Middle legs resemble hind legs. Tarsi of all legs dark brown with black hair. Wing is brown and mottled characteristic of genus, with typical basicosta with sharp projection and costa covered with short black hair without longitudinal groove. Wing with brown patterns (descriptive of species *H. duttoni*) and right-angled thick white line (shaped as “7”) running from vein A1 towards wing margin. Wing vein R4 with long appendix (Fig. 4E, Suppl. material 2: Figure S10D). Halteres yellowish white centrally and progressively darken to light brown and then to darker brown at margin. Stalk of halteres yellowish white.

Abdomen. Abdomen dark brownish black without patterns and with black and white hair and lateral margins almost parallel to each other. Ventrally, abdomen ashy grey with black hair. Median part dark brown with fine white hair (Suppl. material 2: Figure S10A).

#### Haematopota fenestralis

Taxon classificationAnimaliaDipteraTabanidae

Oldroyd, 1952

Fig. 3K

##### Location.

Shimba Hills, Zunguluka, Kenya,

Flies in this species are smaller than *H. duttoni* Newstead, Dutton & Todd (1907) described above.

##### Descriprion.

Head. Head black, broader than long (Suppl. material 2: Figure S11A). Eyes black with colourful bands and separated by wide, more or less square greyish black frons. Frons with short black hair and few white hairs. Frontal callus black and glossy, broad but shorter than and not as convex as in previously described species. Sub callus brownish black and velvety, continuous with frontal callus. Small oval pale black spot medially above frontal callus close to vertex. Antennae brownish grey (tan) with three segments typical of *Haematopota*; scape long and narrower than in *H. duttoni* with black hair, second is globular with anterior thorn-like projection with black hair, flagellum narrow and brown towards base, black annulations with black hair. Second segment of palpus grey with black and white hair, labellum brownish black (Fig. 4F; Suppl. material 2: Figure S11B).

Thorax. Thorax greyish brown with clear thin brown medial stripe extending to posterior end of mesonotum, and brown sublateral stripes, slightly wider than median stripe but only reach half way mesonotum. Posterior mesonotum ashy grey, scutellum greyish brown. Halteres yellowish white medially but progressively darken into brown at periphery. Stalks of halteres yellowish white. Fore tibia slightly swollen (wide) anteriorly with yellowish transverse band towards proximal end with white hair. Fore femur dark brown with dark brown hair and few white hairs. Fore tarsus dark brown with dark brown hair. Hind tibia with two transverse bands, proximally and medially; hind femur dark brown with black and white long hair; hind tibia dark brown with black hair and white bands with white hair. Middle leg looks like hind leg. No hair tufts at postalar tufts. Wing with brown and mottled patterns descriptive of genus. Thick double white streak running across wing apex from anterior, as indicated in black oval in Suppl. material 2: Figure S11C.

Abdomen. Narrow abdomen tapers slightly posteriorly (Suppl. material 2: Figure S11A). Dark brown and even darker on segments 4 to 7 with black and white hair. Ventral abdominal surface ashy grey with white hair; thick shiny brown patch with fine black hair medially on all segments.

### Genus *Chrysops*

Generally, members of this genus are fragile black flies smaller than all the previously described genera. Members have a characteristic black band that transverses the wing from anterior to posterior margin (Fig. 3L).

#### Chrysops distinctipennis

Taxon classificationAnimaliaDipteraTabanidae

Austen, 1906

Fig. 3L

##### Location.

Wakiso, Uganda

##### Descriprion.

Head. Head as wide as thorax with black eyes separated by greyish black frons. Frons widens slightly towards antennae with black and long white standing hair (Suppl. material 2: Figure S13A). Callus black and glossy. Basal callus wider than long and rectangular shaped; upper callus with three black ocelli arranged in triangular pattern (two lateral and one median ocellus). Antennae black, long and slender with black hair. Scape and pedicel equal in length and longer than in *C. brucei* (Suppl. material 2: Figure S13B); flagellum has 4 annulations. Second segment of palpus brownish black, slender with black hair; black labellum.

Thorax. Thorax black with black and white hair, no evident stripes. Tufts of golden yellow hair at postalar callus and at notopleural and humeral lobes (Suppl. material 2: Figure S3B). Halteres black with black stalk. Legs brown with black hair; fore tibia darker brown shade and black distally; tarsus of fore leg black with black hair; tarsi of middle and hind legs brown with black hair. Femurs of all legs black proximally and distally. Clear wing with longitudinal dark brown band that bifurcates into two smaller bands that reach posterior wing margin (Suppl. material 2: Figure S13C).

Abdomen. Abdomen black with black hair and parallel lateral sides; posterior border of each segment distinctly grey. Seventh segment rather rounded (Suppl. material 2: Figure S13C). Ventral surface black with long fine whitish hair.

#### Chrysops brucei

Taxon classificationAnimaliaDipteraTabanidae

Austen, 1907

Fig. 4G

##### Location.

Wakiso, Uganda

##### Descriprion.

Head. Head as wide as thorax with black eyes separated by dark brown frons. Frons widened slightly towards antennae and has black and brown long-standing hair. Callus black and glossy. Lower callus wider than long and oval shaped; upper callus with three ocelli arranged in triangular pattern (two lateral and one median ocellus) (Suppl. material 2: Figure S12A). Antennae black with long black hair. Scape and pedicel almost of equal length although scapes slightly longer. Flagellum equally slender with indistinctly marked annulations. Second segment of the palpus brown, slender with black hair; labellum black (Fig. 4G; Suppl. material 2: Figure S12B).

Thorax. Black thorax with black and brown hair; sub-lateral stripes brown and reach posterior border of mesonotum. Basicosta of wing black with prominent thorn-like projection, while the costa is slender and long without longitudinal groove. Clear wing with longitudinal dark brown band that does not reach posterior margin. Fore leg dark brown with dark brown hair (Suppl. material 2: Figure S12B). Black halteres and stalk.

Abdomen. Abdomen black with black and white hair and parallel lateral sides that taper slightly at fifth segment. Each segment ashy grey at posterior margin with predominantly white hair. Indistinct greyish median stripe. Seventh segment rounded; ventral abdominal surface black with long fine whitish hair.

## Discussion

The aim of this study was to identify, re-describe and document tabanids in East Africa using morphological features and molecular tools and therefore contribute to understanding the diversity of this important group of disease vectors. It is evident that a highly diverse tabanid fauna is prevalent in the areas where the study was carried out.

The specimens in the genus *Atylotus* were initially assigned species names based on their morphological features and were found to differ from those that had been described by Oldroyd (1954). These differences may be accounted for by differences in sampling sites in Ethiopia (Oldroyd 1954) versus Tanzania (Tarangire National Park) and Kenya (Nguruman, southern Kenya) in the current study. These observations may be due to cryptic speciation as was suggested by Schutz et al. (1989). This observation followed a population-genetic study of adult female *Tabanus lineola lineola* Fairchild from New Jersey and Louisiana and determined the coastal and inland “varieties” *T. lineola hinellus* Philip and *T. lineola lineola* Fairchild based on four easily measured morphological characters. In that study, Schutz et al. (1989) found that nine individuals in the study population were genetically distinct from all other specimens and probably represented a third cryptic species. In the current study, the two different *Atylotus* species (*At. nigromaculatus* and *At. diurnus*) clustered together irrespective of their distribution and morphology. These species that were collected in different areas in Kenya (Sampu, Muhaka, Mukinyo) and Tanzania (Sangaiwe, and Poachers Hide) could not be differentiated based on their *COI* barcode sequences, which were nonetheless different from *At. miser* previously sampled in China (Wang et al. 2016). Furthermore, *COI* barcoding in the current study showed that *T. gratus* from Nguruman in Kenya differed from *T. gratus* collected from Tanzania. Morphological differences were also observed where *T. gratus* from Kenya showed a lighter brown abdomen when compared to those from Tanzania.

In addition, *COI* barcoding was able to differentiate *T. taeniola* sensu stricto from its subspecies *T. taeniola variatus*; the later showed some morphological differences (shape of the callus and the patterns on the dorsal surface of the abdomen) and formed a cluster distinct from *T. taeniola*. These observed differences may be due to the sampling locations of the specimen. Similarly, in India, a total of 46 specimens belonging to seven species in four genera in two subfamilies were analysed in a recent study using DNA barcoding; all morphologically identifiable species were successfully discriminated. The study further demonstrated the presence of cryptic species in *Chrysops dispar* and was also able to discriminate closely related species in a “*Tabanus striatus* species complex” that had no stable taxonomic distinguishing characters (Benerjee et al. 2015). Differences in morphological features and DNA barcodes were used as a basis to consider new haplomorphs of deerflies and horseflies that were identified in the study conducted in Canada. Among the specimens, each tabanid species was found to possess distinct sets of *COI* haplotypes which discriminated well among species (Cywinska et al. 2010). Therefore, the authors suggested that *Chrysops montanus* be provisionally differentiated into two haplotypes namely *Ch. montanus* haplomorph 1 and *Ch. montanus* haplomorph 2, each defined by their molecular sequences and by newly discovered differences in structural features near the ocelli. Cryptic species have also been described in other insect species including biting midges in Sweden, where two cryptic species were created within each of the species of *Culicoides obsoletus* and *C. newsteadi* (Diptera: Ceratopogonidae) following the demonstration of a relatively deep intraspecific divergence (Ander et al. 2013). This may be the case with two *T. thoracinus* B sequences obtained in this study, which were morphologically indistinguishable from those in the distinct *T. thoracinus* A clade, but clustered closer to *T. donaldsoni*. Our results demonstrate that morphologically identical *T. thoracinus* could correspond to different species, thus the need to revise the taxonomy of tabanids.

Given the continuous ecosystem with the movement of pastoralists and their livestock across borders, as well as wild fauna, the presence of diverse species of tabanids across the East African region is indicative of the risk for disease transmission. Further study of tabanids in the region is warranted. A better coverage of the tabanids in different ecosystems, including the conservation areas and farming communities, will allow better understanding of the risk of transmission of diseases vectored by these flies in both wild and domestic animals. Such information will also be useful for the ecologist and epidemiologist as expounded by Valentini et al. (2008). The conservation areas of Shimba Hills in Kenya were found to have a more varied range of tabanids despite the fact that the tabanid flies were not the main target of the collection but rather the tsetse fly. Traps routinely used for tsetse flies (NG2G and biconical) (Egri et al. 2013; Allsopp 1984) were used in Kenya and Tanzania and this may have led to non-exhaustive sampling. The varied range may be attributed to by the higher number of tabanids that were collected in the Shimba Hills compared to numbers collected in other study areas. Further surveys should use traps better optimised for tabanids including canopy traps that have been documented to efficiently trap tabanids (Muirhead-Thomson 1991) and have been further improved by addition of carbondioxide (CO_2_) or chemical attractants, e.g. ammonia, phenol, octenol or acetone (Hribar et al. 1992; Mihok et al. 2006; Mihok and Lange 2012). To further enhance the catch of male tabanids, there have been suggestions to supplement the canopy trap with a liquid trap that enhances catches 2.4–8.2 times more tabanids than the canopy trap alone (Egri et al. 2013). The Nzi trap is another option that is used in Africa to capture tabanids; it is simple, economic and efficient (Mihok 2002); it was shown to trap statistically more tabanids than other conventional traps (including NG2G, a triangular, one-winged trap) and equivalent to the canopy trap (Mihok 2002).

Attempts to document Tabanidae in conservation areas in East Africa have been minimal. Nonetheless, tabanids of the Afrotropical region of southern Africa have been recently re-described, including discussion on their biology, immature stages, economic importance, and classification with a resultant identification key of Afrotropical Tabanidae (Chainey 2017). Studies exploring tabanid flies in Muhale Mountains National Park in Tanzania have similarly shown a sizable number of species (17) that fed on chimpanzees (Sasaki and Nishida 1999; Sasaki 2005). One of the species in Muhale (*T. gratus*) was also found in Tarangire National Park in the current study; all the other species were different. The difference may be due to the different techniques used (NG2G trap in Tarangire versus Nzi trap in Muhale) but may also be attributed to the differences in the climate and vegetation cover of the two sites in Tanzania that were a forested mountain and a thickly and mixed woodland. Inevitably, there is need to understand the influence of climate on the prevalence of vectors thus be able to forecast vector-borne disease outbreaks in a given ecosystem (Gubler 2008).

There are new technologies that can be used to enhance the understanding of relationships between the environment and vector as well as risk of emerging vector-borne diseases. These tools continuously capture and analyse a wide range of environmental data including weather, land cover and use, water and atmospheric conditions (Ostfeld et al. 2005) that can be applied in research on vector-borne diseases (Beck et al. 2000). Using new statistical methods, spatial patterns in this data can reveal relationships between the vector and vector-borne pathogen (cause) and disease (effect) (Hay et al. 2000, 2007).

In East Africa, inter-organismic relationship studies using DNA barcoding have been done to identify the source(s) of blood meal for tsetse flies (genus *Glossina*) and reported diverse source of blood meal including wild mammals (buffalo, giraffe, warthog, elephant and spotted hyena), livestock (cattle). These approaches have similarly been used to identify the diverse blood meal hosts of mosquitoes (Lutomiah et al. 2014; Omondi et al. 2015). The authors of these studies note that these results are useful in designing effective vector control strategies based on their host preferences in the various intervention areas. Among others, these studies strongly suggest that DNA barcoding is a promising tool for diversity studies, phylogeographic analyses as well as evolutionary studies of arthropods specifically as vectors, and thus could provide a globally important tool for controlling pests. However, to efficiently use these novel tools, there is a need for sustained capacity building in public health as well as in environmental/climatic studies and to understand how the two disciplines can be merged in vector-borne disease research (Fish 2002).

## Conclusion

This is the first sudy that describes in detail the morphology of diverse tabanids from various locations in Kenya, Tanzania and Uganda. The list of tabanids presented in this paper is largely incomplete given the limited study sites and duration of sampling. Thus, further investigation is required to comprehend the whole fauna of tabanid flies and their habitat. Wider surveys that integrate climatic factors, pathogens carried by the tabanids, blood meal sources for the flies, as well as the effect of human activity on their distribution are necessary if the diversity, prevalence and role of tabanids in disease transmission are to be better understood. This will, in turn, enable the instigation of more efficient control measures against these neglected vectors. The findings from the hand-full of sampling sites indicate a need to further investigate neglected insects on a larger scale to establish a concrete tabanid checklist in East Africa.

## Supplementary Material

XML Treatment for Ancala fasciata

XML Treatment for Tabanus donaldsoni

XML Treatment for Tabanus gratus

XML Treatment for Tabanus guineensis

XML Treatment for Tabanus taeniola

XML Treatment for Tabanus taeniola variatus

XML Treatment for Tabanus thoracinus

XML Treatment for Atylotus nigromaculatus

XML Treatment for Atylotus diurnus

XML Treatment for Haematopota duttoni

XML Treatment for Haematopota fenestralis

XML Treatment for Chrysops distinctipennis

XML Treatment for Chrysops brucei

## Supplementary materials

Supplementary material 1
Tabanidae collected (n) from Uganda, Kenya and Tanzania This is an open access article distributed under the terms of the Creative Commons Attribution License (CC BY 4.0), which permits unrestricted use, distribution, and reproduction in any medium, provided the original author and source are credited.Claire M. Mugasa, Jandouwe Villinger, Joseph Gitau, Nelly Ndungu, Marc Ciosi, Daniel Masiga

Supplementary material 2 Supplementary figures S1-S13 This is an open access article distributed under the terms of the Creative Commons Attribution License (CC BY 4.0), which permits unrestricted use, distribution, and reproduction in any medium, provided the original author and source are credited.Claire M. Mugasa, Jandouwe Villinger, Joseph Gitau, Nelly Ndungu, Marc Ciosi, Daniel Masiga
