# Supplementary material for: Morphological re-description and molecular identification of Tabanidae (Diptera) in East Africa
Source: Zookeys. 2018 Jun 26;(769):117–44. doi: 10.3897/zookeys.769.21144 (PMC6030178; doi:10.3897/zookeys.769.21144)
Supplement: Supplementary material 1 — Tabanidae collected (n) from Uganda, Kenya and Tanzania [file zookeys-769-117-s001.docx]

**Supplementary Table 1. *Tabanidae* collected (n) from Uganda, Kenya and Tanzania**

| **Subfamily** | **Genus** | **Species** | **Kenya (n)** | **Tanzania**  **(n)** | **Uganda**  **(n)** |
| --- | --- | --- | --- | --- | --- |
| Tabaninae | *Ancala* | *A. fasciata* | 0 | 0 | 50 |
|  | *Tabanus* | *T. thoracinus* | 5 | 0 | 977 |
|  |  | *T. donaldsoni* | 3 | 0 | 0 |
|  |  | *T. taeniola* | ~2000 | ~2000 | 0 |
|  |  | *T. taeniola variatus* | 0 | 14 | 0 |
|  |  | *T. guineensis* | 2 | 0 | 0 |
|  |  | *T. gratus* | 1 | 6 | 0 |
|  | *Atylotus* | *At. nigromaculatus* | 321 | 288 | 0 |
|  |  | *At. diurnus* | 2 | 1 | 0 |
|  | *Haematopota* | *H. duttoni* | 16 | 0 | 0 |
|  |  | *H. fenestralis* | 5 | 0 | 0 |
| Chrysopsinae | *Chrysops* | *C. brucei* | 0 | 0 | 2 |
|  |  | *C. distinctipennis* | 0 | 0 | 11 |
