# Supplementary material for: Morphological re-description and molecular identification of Tabanidae (Diptera) in East Africa
Source: Zookeys. 2018 Jun 26;(769):117–44. doi: 10.3897/zookeys.769.21144 (PMC6030178; doi:10.3897/zookeys.769.21144)
Supplement: Supplementary material 2 — Supplementary figures S1-S13 [file zookeys-769-117-s002.pdf]

Supplementary Figures

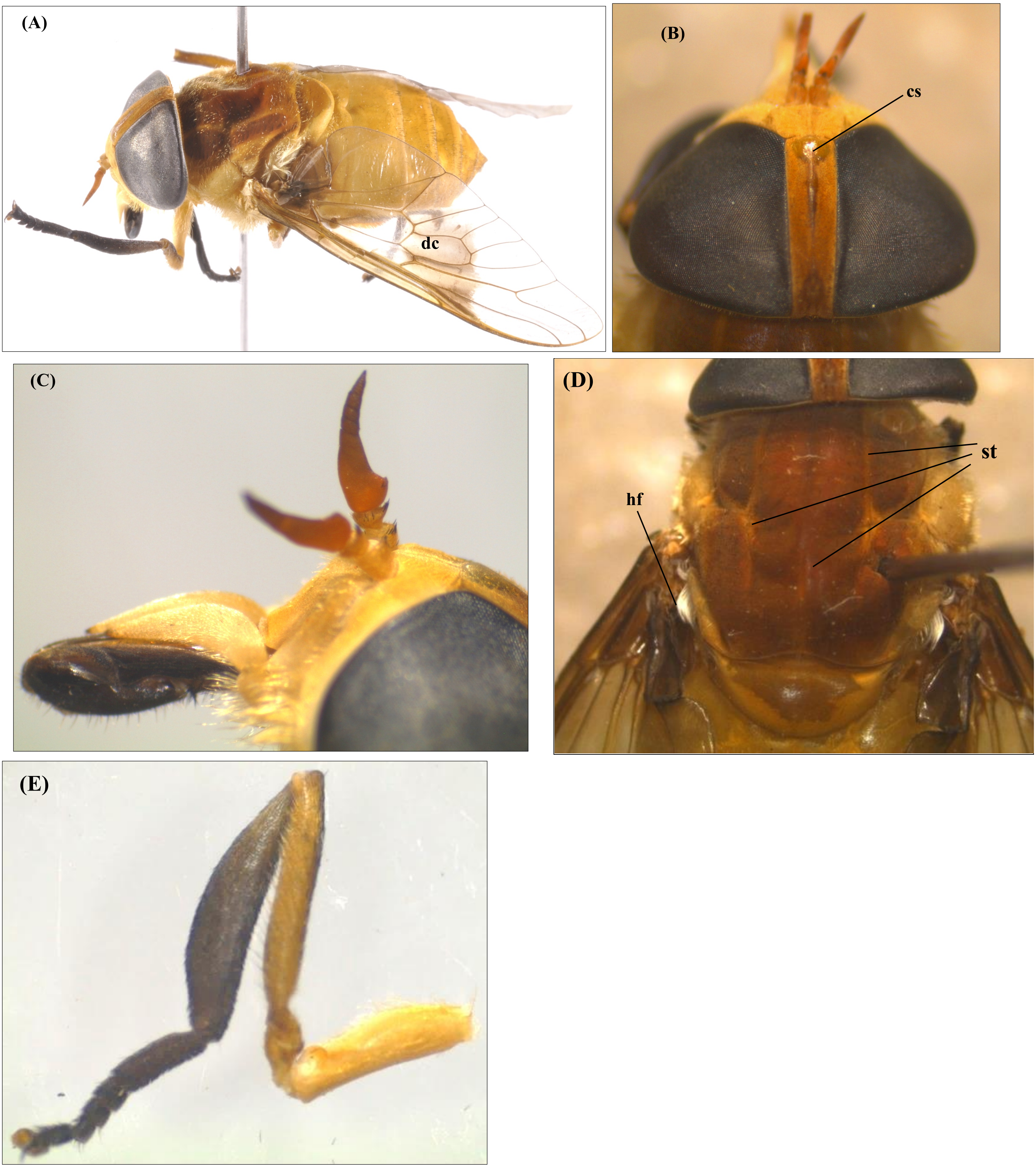

Figure S1: Distinctive morphological characteristics of *Ancala fasciata*. (A) Dorso-lateral view of showing uniformly brown discal cell (dc); (B) Brown frons and callus (cs); (C) Antenna and mouth parts; (D) brown thorax with brownish-black patterns with stripes (st) that may be distinct or not, distinct white hair tufts (hf) at the wing base (E) All black tibia of *Ancala fasciata*.

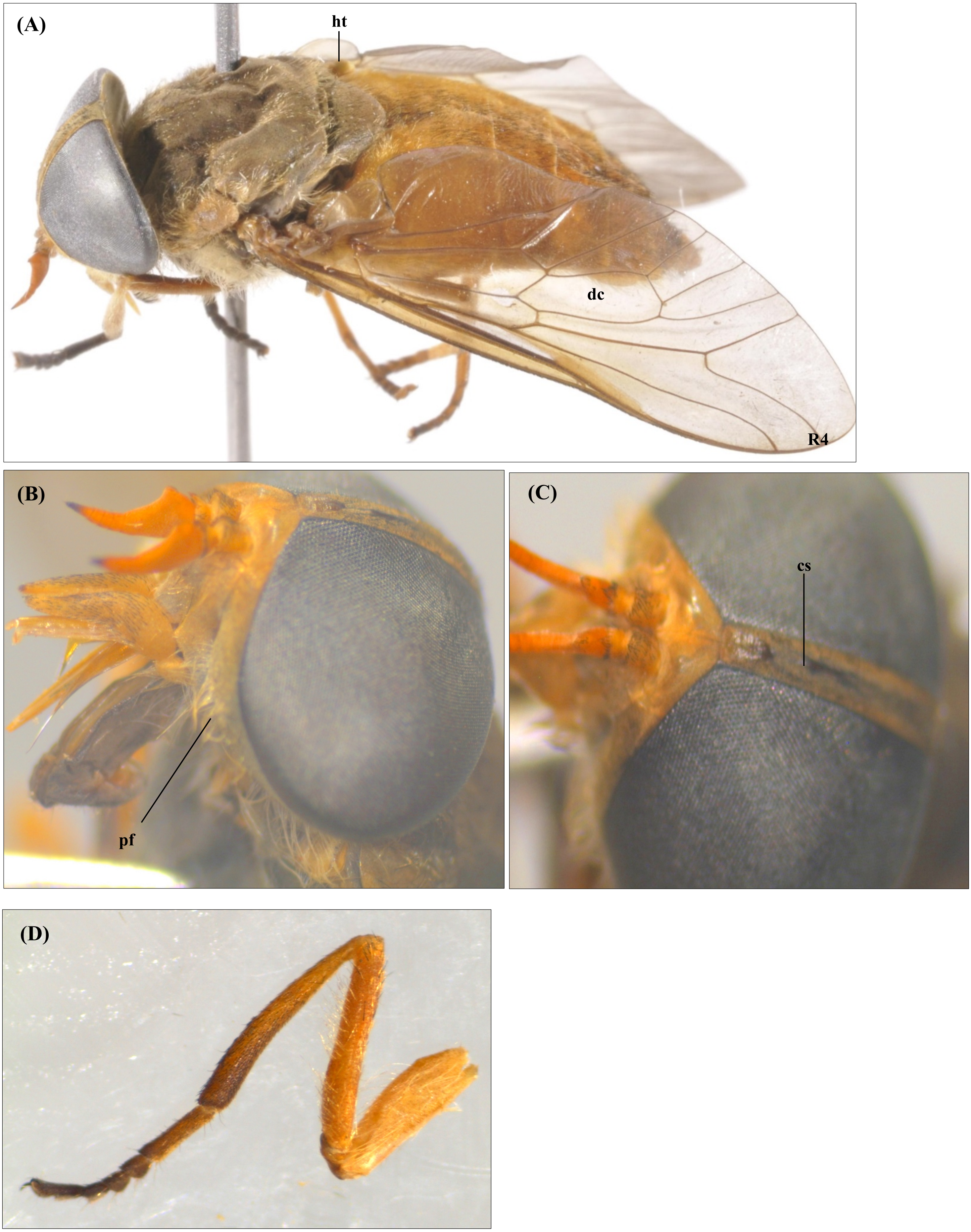

Figure S2: Distinctive morphological characteristics of *Tabanus thoracinus.* (A) dorso-lateral view of a no[illegible] patterned brown body, black eyes in preserved specimen, clear wing and clear discal cell (dc) and brown haltare (h[illegible] vein R4 has no appendix; (B) antennae mouth parts and parafacial hairs (pf); (C) narrow frons and dark brown-bla[illegible] callus (cs); (D) all brown fore leg.

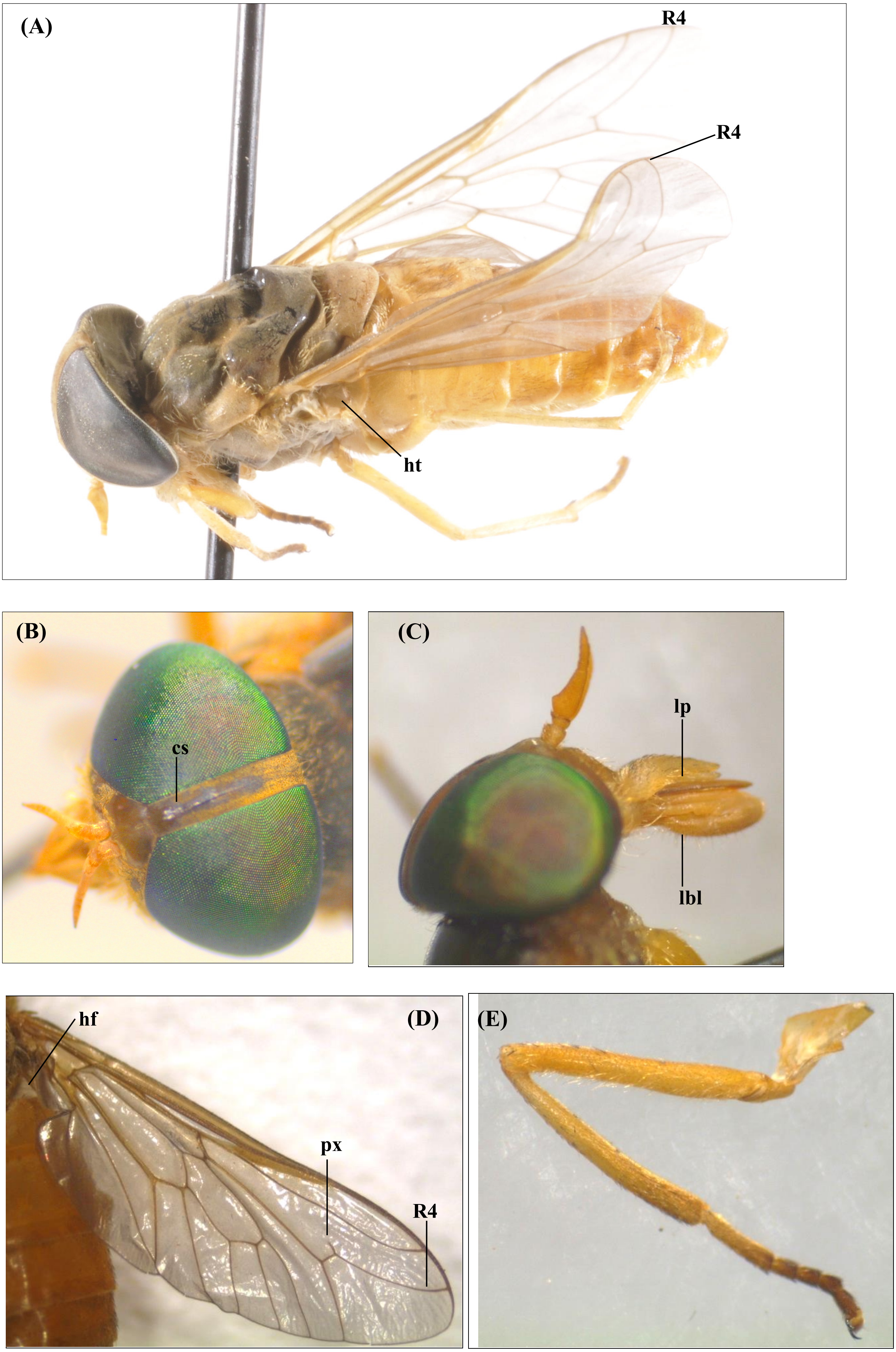

Figure S3: Distinctive morphological characteristics of *Tabanus donaldsoni*. (A) dorso-lateral view showing a plain golden-brown abdomen, fore and hind legs are golden brown; (B) dorsal view of the head showing green eyes ir freshly collected specimen with a brown callus; (C) morphology of antennae and mouth parts; (D) clear wing with distinct R4 appendix (px) and indistict hair tuft t the base; (E) all brown fore leg.

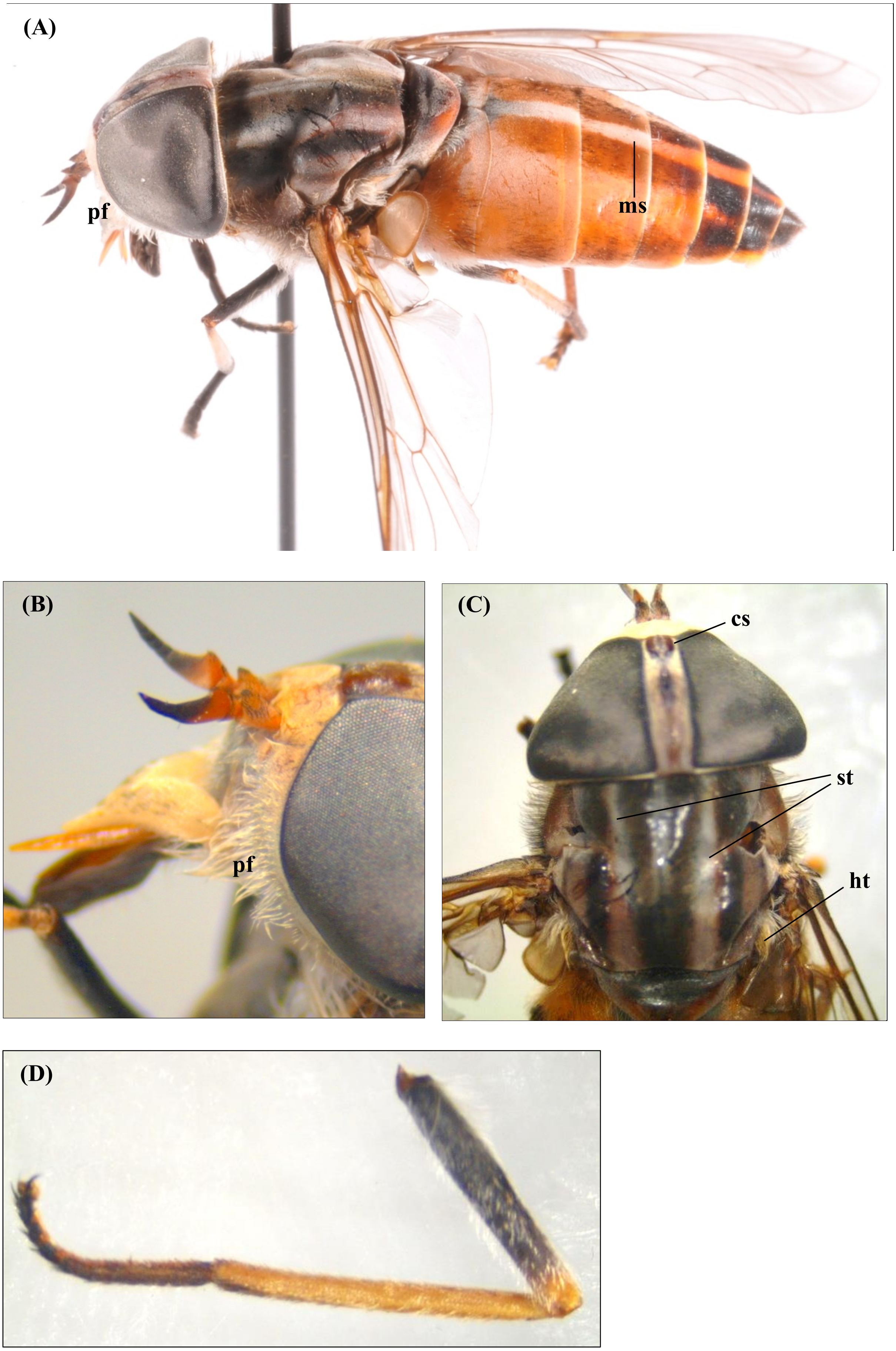

Figure S4: Distinctive morphological characteristics of *Tabanus taeniola*. (A) dorso-medial view median s stri (ms); (B) head showing long white para-facial hairs, brown antennae with black hairs, palpus has numerous white ar few black hairs, the labellum is black; (C) frons with bell- shaped callus (cs), distinct thick grey thorax stripes (st) ar haltares (ht); (D) hind leg (partial).

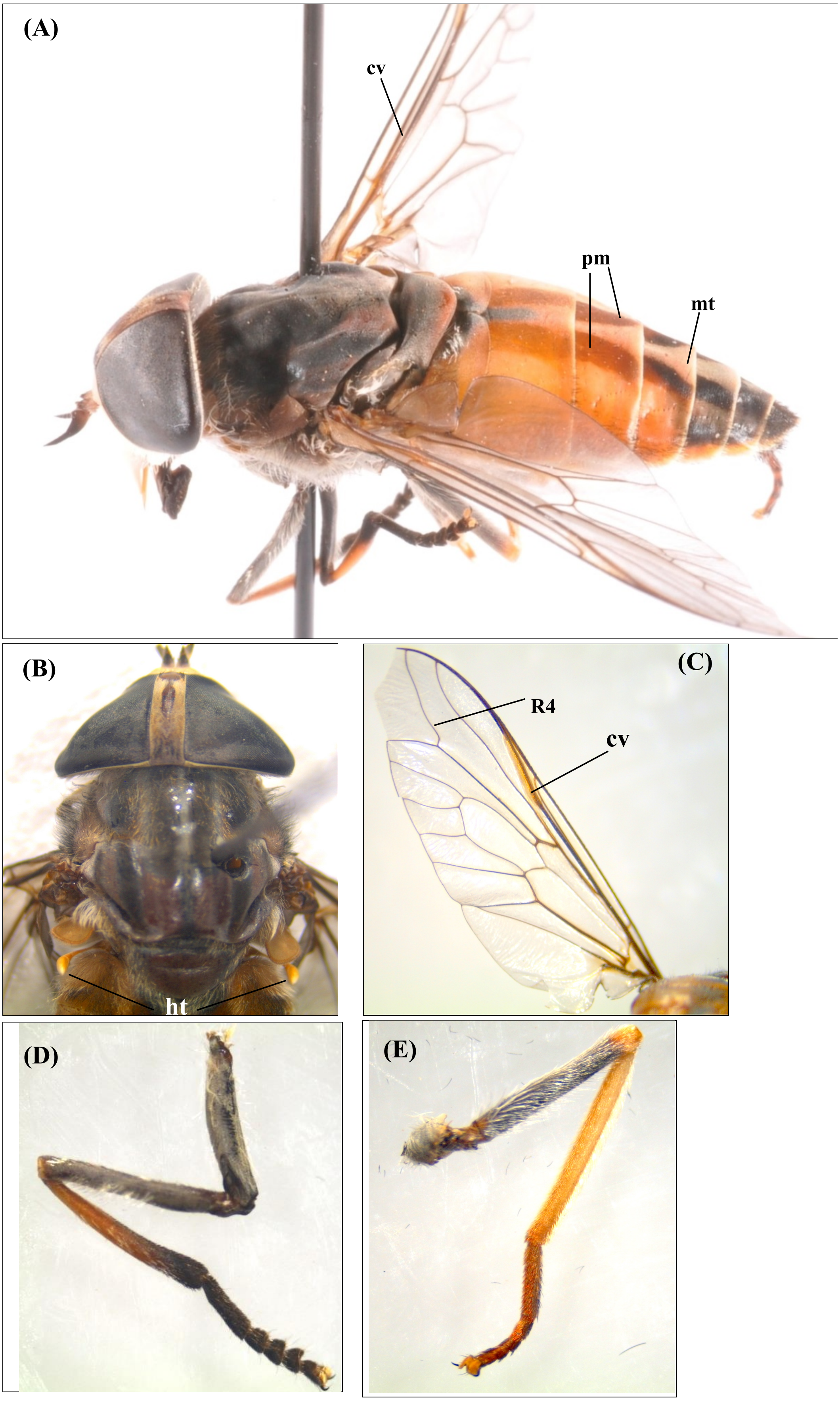

Figure S5: Distinctive morphological characteristics of *Tabanus taeniola variatus*. (A) dorso-medial view showin[illegible] the medial triangles (mt) and peri-median bands (pm);(B) dorsal view of head and thorax; (C) clear wing, brown costa[illegible] veins (cv); (D) fore leg; (E) hind leg (part).

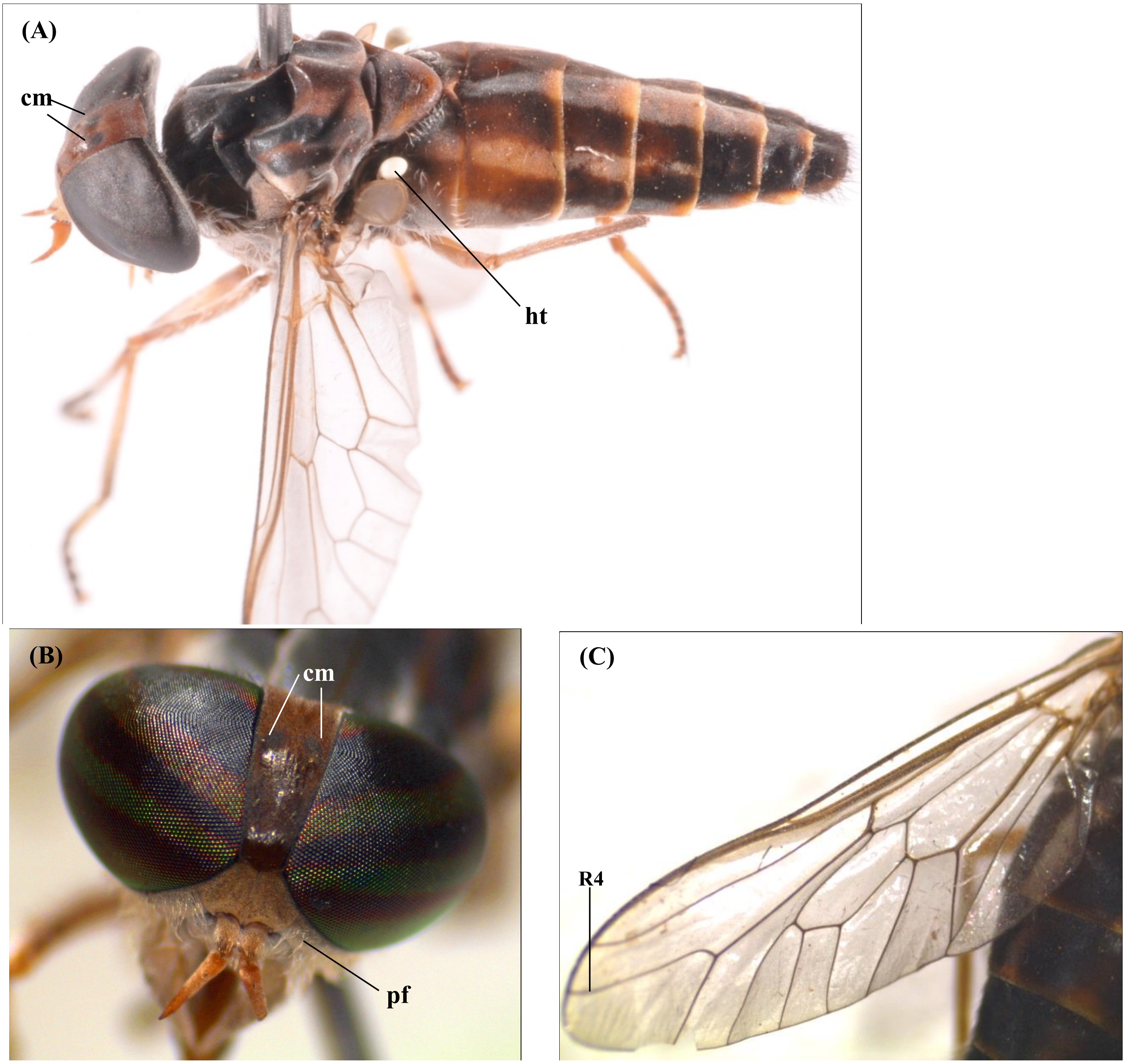

Figure S6: Distinctive morphological characteristics of *Tabanus gratus*. (A) dorso-medial view showing the thic white abdominal bands, white haltares (ht); (B) black eyes with green bands, a callus with thick comma shaped blac shade (cm) besides the callus; (C) clear wing, the R4 has no appendix.

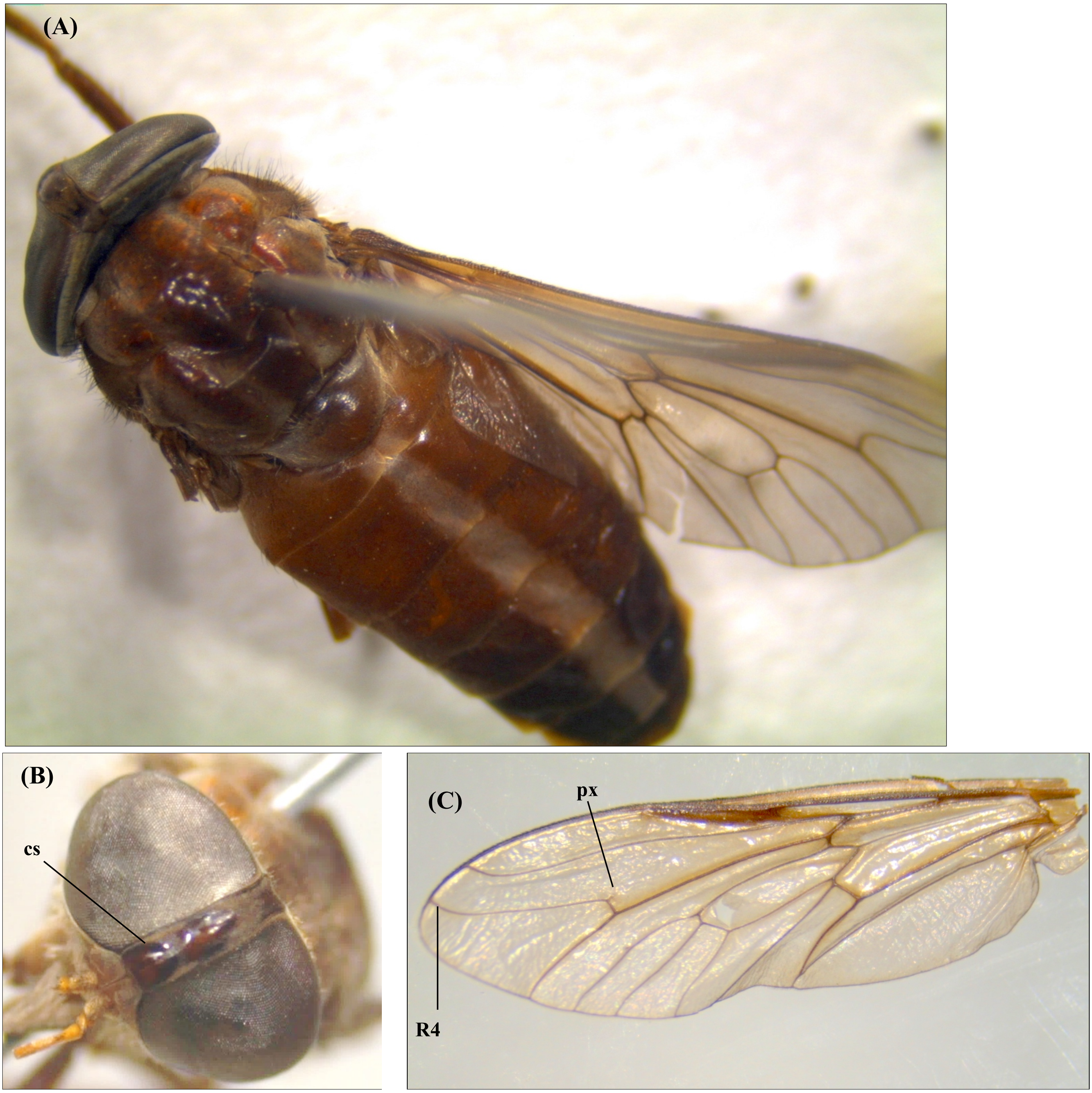

Figure S7: Distinctive morphological characteristics of *Tabanus guineensis.* (A) Dorsal view showing the thic white band running medially down the abdomen; (B) black eyes and thick dark brown bi-partite callus (cs); (C) cle wing with short R4 appendix (px).

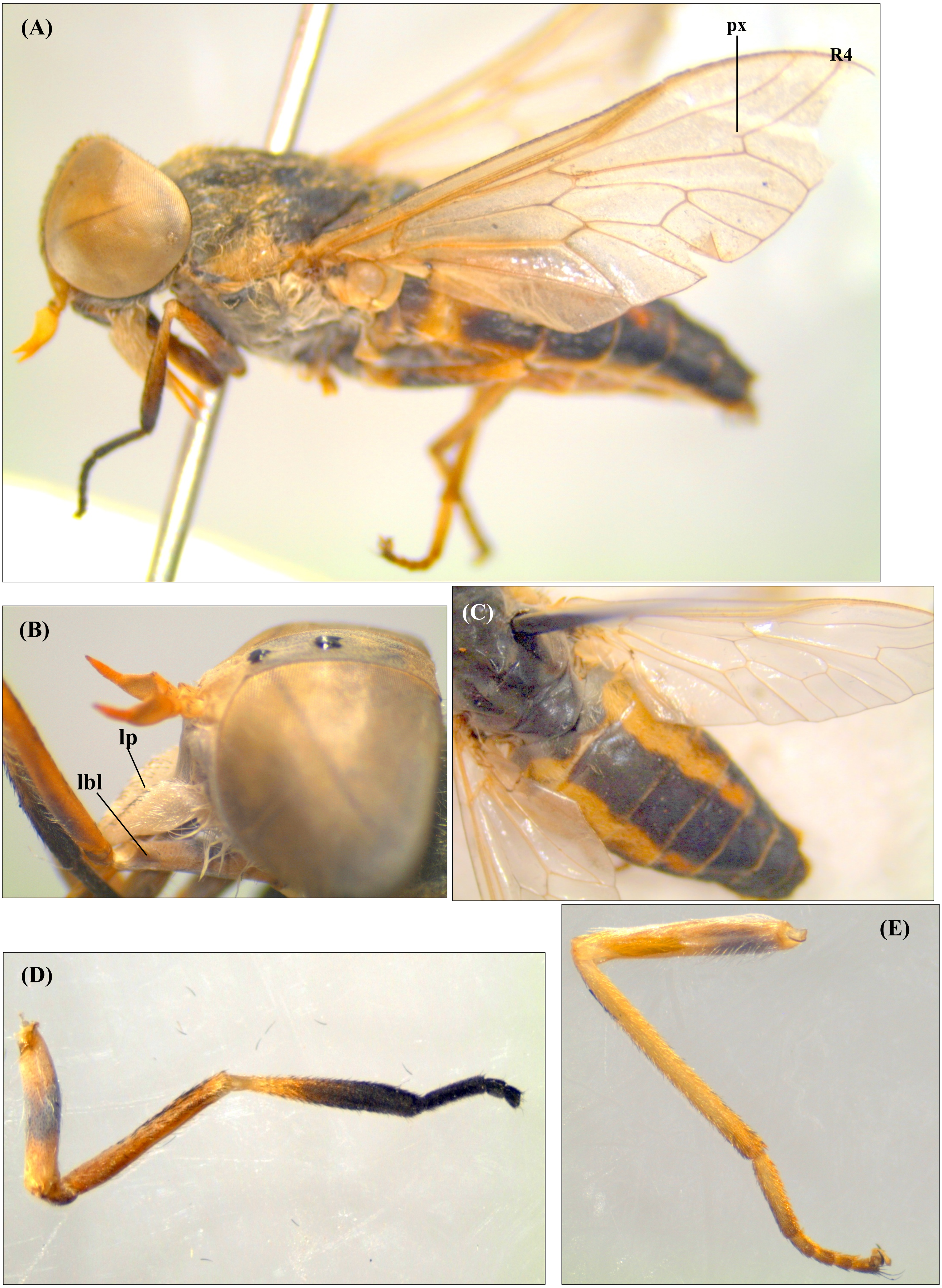

Figure S8: Distinctive morphological characteristics of *Atylotus nigromaculatus*. (A) dorso-lateral view, clear wir with appendix on R4; (B) pale golden brown and have a thin black horizontal line, golden brown antennae brow labellum (lbl); (C) black median abdominal band; (D) yellow anterior third of fore tibia; (E) light brown hind le (part).

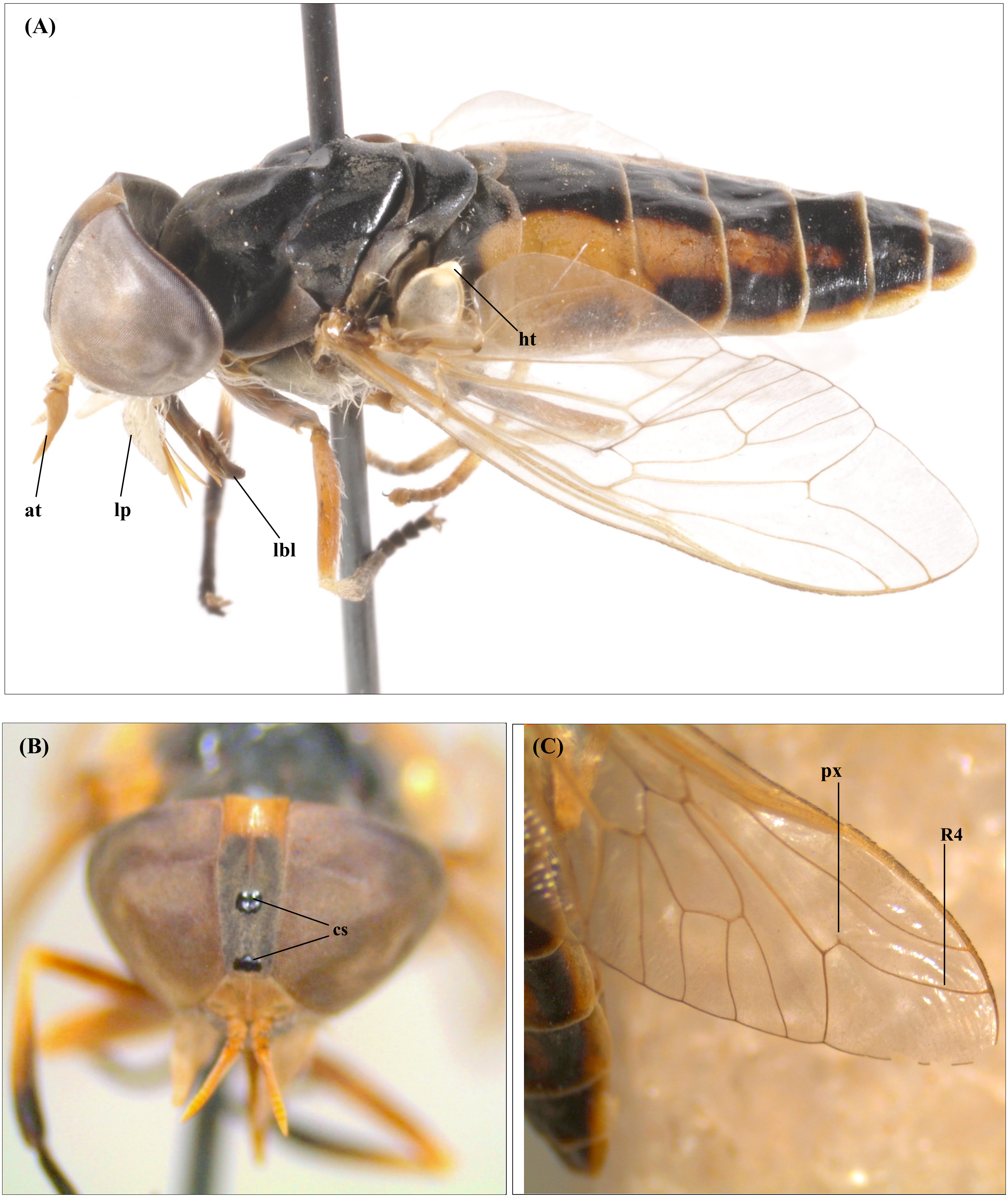

Figure S9: Distinctive morphological characteristics of *Atylotus diurnus*. (A) Dorso-medial view showing the blac medial abdominal band and black thorax and white haltares (ht); medially are the antennae (at), white palpus (lp) ar dark brown labellum (lbl) (B) frons and small black upper and lower callus (cs); (C) clear wing with indistin appendix (px) on R4.

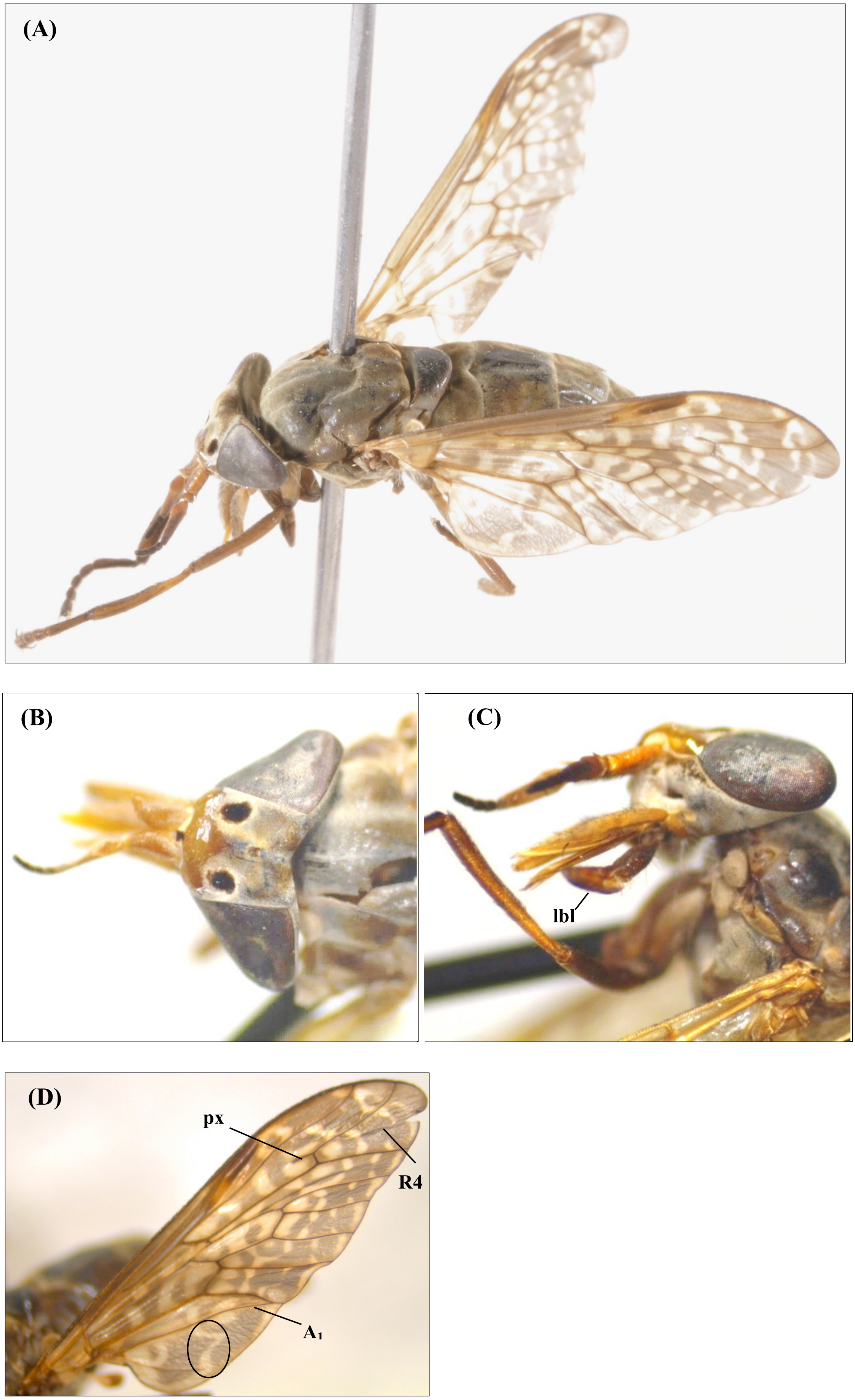

Figure S10: Distinctive morphological characteristics of *Haematopota duttoni*. (A) dorso-lateral view showin wing morphology and uni-coloured brown legs; (B) frons and brown shiny callus; (C) antennae and mouth parts; (I mottled wing with a right angled white thick line (circled) between vein A_1 and the wing margin, the R4 has a lor appendix (px).

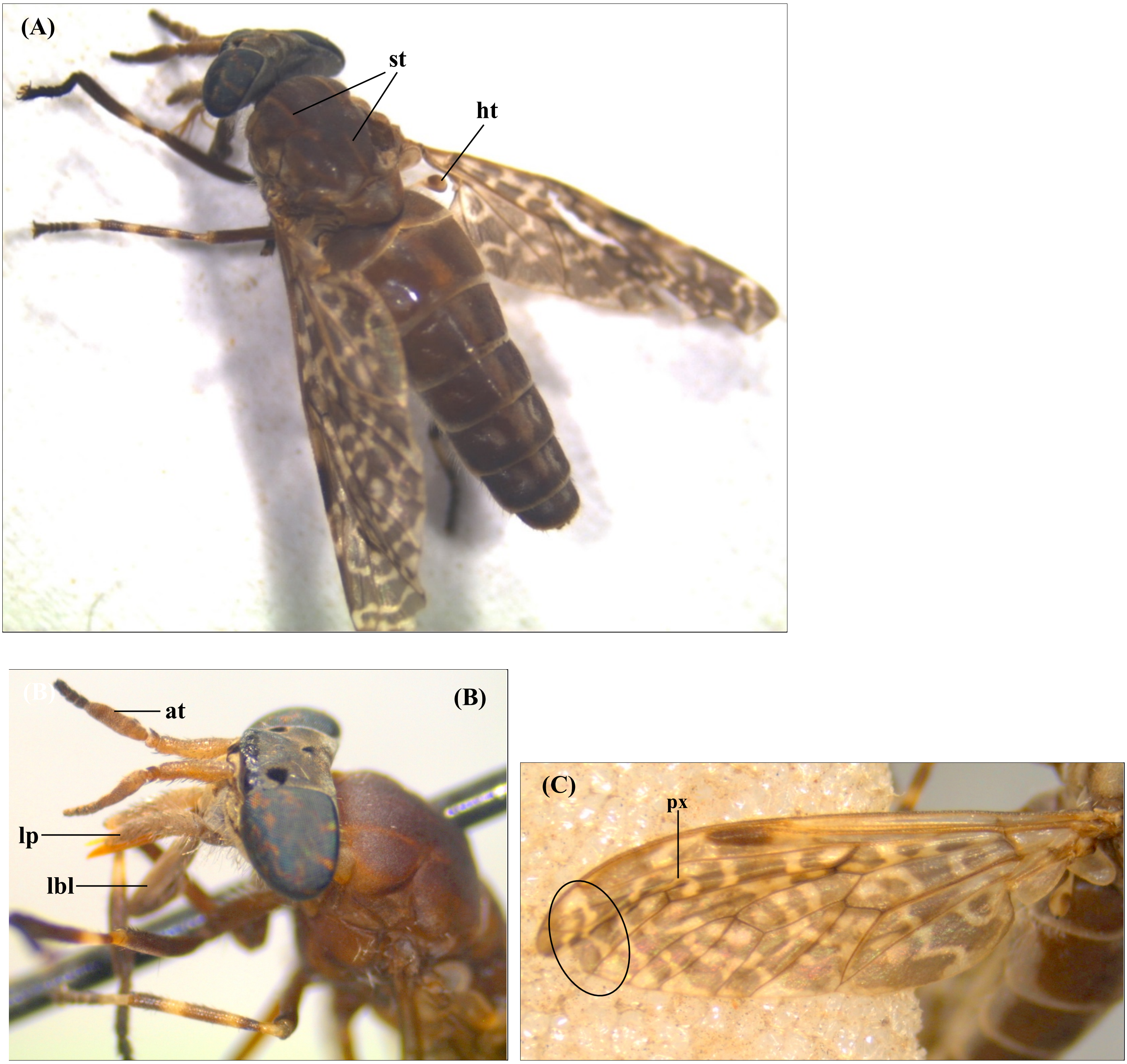

Figure S11: Distinctive morphological characteristics of *Haematopota fenestralis.* (A) dorsal view ; (B) frons, black callus and banded eyes, antennae and characteristic banded leg; (c) characteristic mottled wing and venation between with a thick double white streak running across the wing apex from anterior (in the black oval), and a long appendix on wing vein R4.

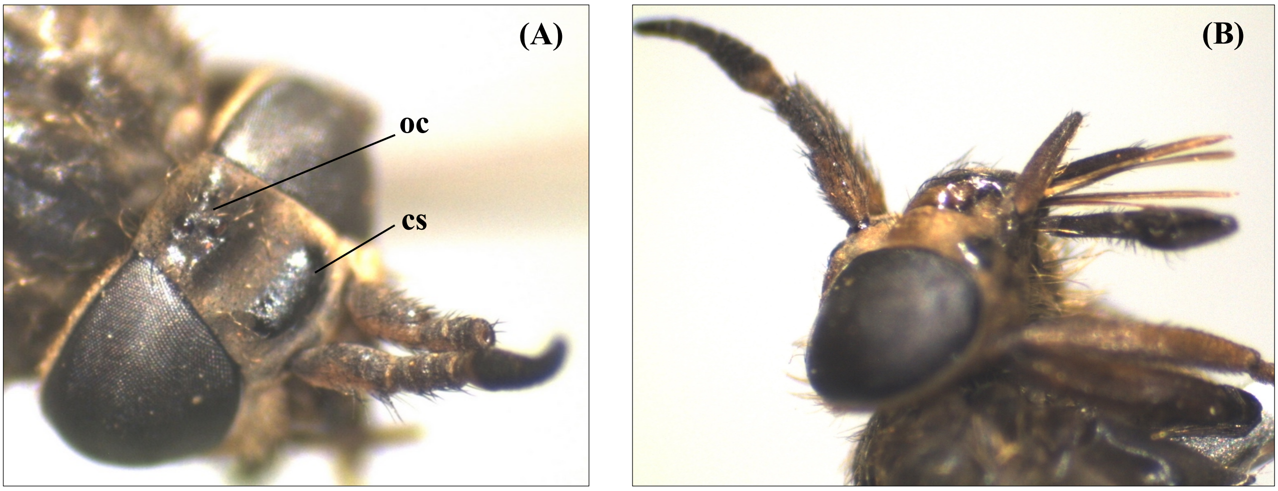

Figure S12: Distinctive morphological characteristics of *Chrysops brucei*. (A) Dorsal view revealing the abdome[illegible] and transverse black band on the wings; three round black ocelli (oc) and lower callus (cs); (B) antenna, black mou[illegible] parts and brown legs (partial).

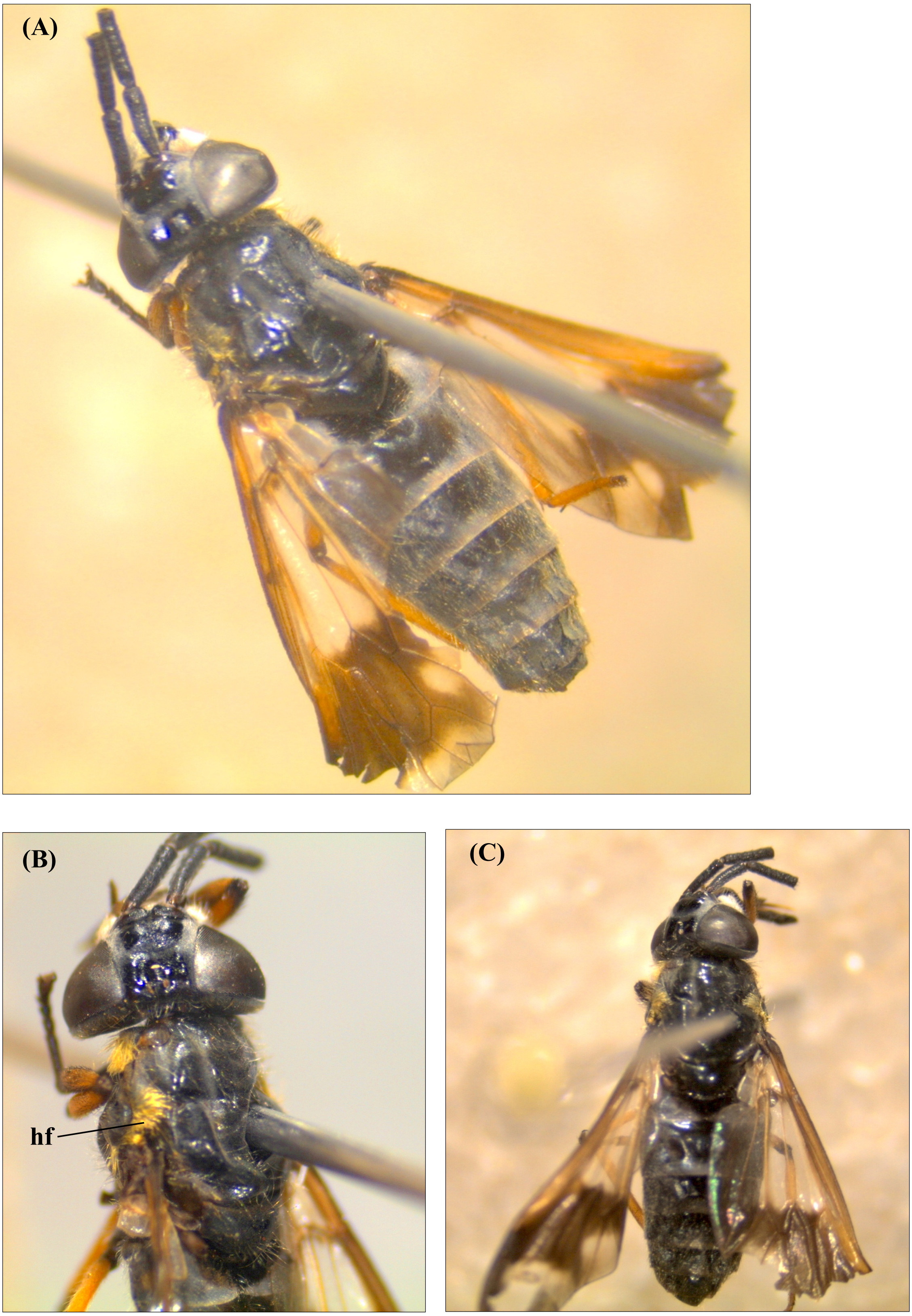

Figure S13: Distinctive morphological characteristics of *Chrysops distinctipennis*. (A) Dorsal view showing the slender tapering black abdomen (the third antennal segment broke off during handling); (B) golden yellow hair tufts (hf) on the postalar callus (C) wing band bifurcates to the posterior margin.
